# Striated muscle: an inadequate soil for cancers

**DOI:** 10.1007/s10555-024-10199-2

**Published:** 2024-07-12

**Authors:** Alastair A. E. Saunders, Rachel E. Thomson, Craig A. Goodman, Robin L. Anderson, Paul Gregorevic

**Affiliations:** 1 Centre for Muscle Research, and Department of Anatomy and Physiology, The University of Melbourne, Parkville, Victoria 3010 Australia; 2 Metastasis Research Laboratory, Olivia Newton-John Cancer Research Institute, Heidelberg, Victoria Australia; 3 School of Cancer Medicine, La Trobe University, Bundoora, Victoria Australia; 4 Department of Clinical Pathology, The University of Melbourne, Parkville, Victoria Australia; 5 Peter MacCallum Cancer Centre, Parkville, Victoria Australia; 6 Department of Neurology, The University of Washington School of Medicine, Seattle, WA USA

**Keywords:** Cancer, Metastasis, Skeletal muscle, Cardiac muscle, Striated muscle

## Abstract

Many organs of the body are susceptible to cancer development. However, striated muscles—which include skeletal and cardiac muscles—are rarely the sites of primary cancers. Most deaths from cancer arise due to complications associated with the development of secondary metastatic tumours, for which there are few effective therapies. However, as with primary cancers, the establishment of metastatic tumours in striated muscle accounts for a disproportionately small fraction of secondary tumours, relative to the proportion of body composition. Examining why primary and metastatic cancers are comparatively rare in striated muscle presents an opportunity to better understand mechanisms that can influence cancer cell biology. To gain insights into the incidence and distribution of muscle metastases, this review presents a definitive summary of the 210 case studies of metastasis in muscle published since 2010. To examine why metastases rarely form in muscles, this review considers the mechanisms currently proposed to render muscle an inhospitable environment for cancers. The “seed and soil” hypothesis proposes that tissues’ differences in susceptibility to metastatic colonization are due to differing host microenvironments that promote or suppress metastatic growth to varying degrees. As such, the “soil” within muscle may not be conducive to cancer growth. Gaining a greater understanding of the mechanisms that underpin the resistance of muscles to cancer may provide new insights into mechanisms of tumour growth and progression, and offer opportunities to leverage insights into the development of interventions with the potential to inhibit metastasis in susceptible tissues.

**Supplementary Information:**

The online version contains supplementary material available at 10.1007/s10555-024-10199-2.

## Introduction

Tumour cells frequently disseminate from primary sites *via* the bloodstream and lymphatic network, to establish secondary tumours in other organs. However, differences exist between tissue sites and types of primary cancers which influence where metastatic tumours are observed. Common sites of metastasis include lymph nodes, lung, liver, bone, and brain [1]. Two theories have been proposed to explain the non-random pattern of metastatic disease—Stephen Paget’s seed and soil hypothesis, where the disseminated cell will only grow in a supportive microenvironment [2], and James Ewing’s vascular flow hypothesis, where cells are led to tissues by lymphatic or circulatory flow patterns [3]. Observations reported to date suggest that both hypotheses are correct, depending on the type of primary tumour and the sites of colonisation.

Striated muscles are striking as sites of comparatively infrequent primary and secondary cancers. Striated muscle tissues (which include skeletal and cardiac muscles) contain repeated functional units called sarcomeres, with the primary role of providing movement for posture, motion, respiration, communication (in the case of skeletal muscle), and blood circulation (*via* cardiac muscle function). There are examples of primary cancers of skeletal muscle, the most common being rhabdomyosarcoma, which usually occurs in children [4]. However, although considered cancers of the skeletal muscle lineage, rhabdomyosarcomas are typically found in non-muscle sites. Furthermore, although skeletal muscles comprise 30–40% of the body mass of a human [5] and have a rich blood supply [6], they are rarely the host of secondary cancers [4, 7–11]. Similarly, primary cancers of the myocardium (heart muscles) are extremely rare in adults [12, 13], with the rarity of myocardial metastases providing a particularly interesting example given the extensive perfusion of the coronary vasculature.

As metastases are the cause of most cancer-related deaths, an understanding of the factors that result in an organ being susceptible or resistant to metastatic tumour growth is a compelling avenue for research. The literature offers several perspectives as to why muscles are seemingly resistant to metastasis [14–16]. These include the concept that blood flow to muscle may prevent metastatic seeding [17]; that muscle motion destroys cancer cells [18]; that cancer cell proliferation is restricted as a consequence of their fusion with post-mitotic muscle fibres [19]; that secreted factors produced by muscle prevent cancer growth [20, 21], and that the metabolic properties of muscle prevent metastatic outgrowth [22]. In this review, we have sought to summarise the incidence of primary and secondary tumours in striated muscle and discuss the proposed mechanisms that result in a lack of tumours in this tissue. Gaining a greater understanding of the mechanisms that render a tissue type resistant to tumour growth may help to guide the development of new therapies for metastatic disease.

## Incidence of primary striated muscle cancers

Sarcomas can form in muscle, fat, or connective tissue and account for approximately 1% of all adult malignancies [23]. An analysis of over 9 million cancer cases in the USA, that used the definition of 15 new cases per 100,000 of the population to define cancers as “rare”, reported that soft tissue sarcomas occurred at a rate of 3.6 per 100,000 [24]. This comparatively infrequent incidence of primary cancers in muscle is in contrast with cancers of the breast, prostate, lung, colon, ovary, and skin, that were all defined as “common” cancers with more than 15 cases per 100,000 people [24]. Another study that stratified the data by sex found that connective and soft tissue cancers were equally rare in males and females, making up 0.9% and 0.8% of cancers respectively [25]. Rhabdomyosarcoma, the most common soft tissue tumour in children and adolescents makes up approximately 1% of all adult malignancies, which is markedly lower than malignancies in other organs, such as the lung (27.66%), colon (26.71%), breast (24.17%), prostate (17.39%), stomach (15.59%), bladder (6.52%), and liver (6.51%) and that account for most cancer diagnoses [26]. However, it is important to note that not all reported soft tissue sarcomas are intramuscular, as they are most frequently found in sites other than muscle, further highlighting the comparative rarity of skeletal muscle primary cancers. Primary cancers in cardiac muscle occur even less frequently than those in skeletal muscle, being reported to occur in 1.38 out of every 100,000 individuals [27]. Importantly, 90.5% of these cardiac tumours are benign [27]. Autopsy-based studies have shown that the frequency of primary cardiac tumours ranges from 0.02 to 0.056% of autopsy cases. The scarcity of papers that distinguish between intrinsic tumours of cardiac muscle and those that have invaded the heart means that the exact incidence of myocardial cancers is difficult to determine accurately. In summary, reports published to date clearly demonstrate that primary tumours of striated muscles are rare, which is striking given the proportion of body mass typically comprised of muscle, and in the case of skeletal muscles, also includes significant populations of adult muscle stem cells that retain proliferative potential throughout life. The rarity of cancer incidence in striated muscle has fostered interest in underlying mechanisms, which are considered below.

## Proposed mechanisms protecting muscle from primary cancers

### Initiation of primary muscle cancers

Understanding the factors that contribute to the initiation of comparatively rare primary cancers in muscle may reveal tissue-specific environmental cues that could otherwise suppress tumourigenesis. Rhabdomyosarcoma is a soft-tissue sarcoma which resembles undifferentiated skeletal muscle cells [28]. Alveolar rhabdomyosarcoma (ARMS)—an invasive subtype of rhabdomyosarcoma—is characterised by fusion of Pax3 or Pax7 genes on chromosome 1 and 2, respectively, with the FOXO1 gene; which encodes Pax3-FOXO1 and Pax7-FOXO1 fusion proteins [29]. These genetic events result in altered regulation of proliferation, apoptosis, and differentiation which can support tumourigenesis. Embryonic rhabdomyosarcoma (ERMS) does not show any gene fusion but is typically caused by copy number alterations of genes such as TP53 and RAS family genes.

The use of a kRAS-driven zebrafish model of ERMS found that cancer initiation within the musculature occurred at a higher rate in tp53^-/-^ fish, suggesting a role for the p53 pathway in suppressing rhabdomyosarcoma initiation [30]. In support, another study showed that a loss of the KMT3b gene (encoding Nuclear Receptor Binding SET Domain Protein 1) can cause initiation of rhabdomyosarcoma through an aberration of cellular senescence and cell cycle arrest in muscle stem cells through inactivation of p53 [31]. Rhabdomyosarcoma cells display a phenotype reminiscent of an immature myoblast, remaining somewhat resistant to undergoing differentiation into adult skeletal muscle fibres. Inhibited skeletal muscle cell differentiation can, in part, be recapitulated by constitutive expression of c-MYB [32]. Suppression of c-MYB in rhabdomyosarcoma cells was observed to result in reduced tumour engraftment success and a slowing of tumour growth in the initial stages of tumour formation [33]. MYB and MYC expression is implicated in tumourigenesis through enforcing DNA replication [34, 35]. Hippo signalling has also been implicated in rhabdomyosarcoma tumourigenesis, with elevation of the transcriptional regulator, Yes-associated protein (YAP), observed in rhabdomyosarcomas [36]. Moreover, experimental upregulation of YAP activity in activated, but not quiescent, skeletal muscle satellite cells led to hallmarks of ERMS in mouse models [37]. Additionally, others have shown that experimental upregulation of YAP activity in adult skeletal muscle fibres in mice did not recapitulate the ERMS phenotype [38]. These findings collectively demonstrate that the transcriptional programs of quiescent muscle satellite cells and post-mitotic skeletal muscle fibres also contribute to the cells’ relative resistance to oncogenic induction.

### Potential mechanisms that deter primary muscle cancer initiation

Adult skeletal muscle fibres are post-mitotic, with little cell turnover in an uninjured state [39]. However, muscles also contain a significant population of myogenic satellite cells that possess remarkable proliferative potential that enables muscle regeneration following episodes of muscle fibre damage [40]. In uninjured muscles, these satellite cell populations remain largely quiescent [41]. The post-mitotic features of muscle fibres and the quiescent phenotype of muscle satellite cells, unless challenged, have led to suggestions that the adult skeletal muscle environment may somehow limit the mitotic activity of cells with proliferative potential, such as primary cancers. It is possible that contact inhibition as a consequence of interaction with muscle fibres or the cues that muscle fibres release to keep satellite cells quiescent also help to keep cancer cells quiescent [42]. Of note, the risk of cancer occurrence by tissue has been correlated with the number of stem cell divisions that take place to maintain a tissue over its lifetime [43]. However, there is no experimental evidence in muscle that tests this correlation.

Recently, it was proposed that the microenvironment within the heart may prevent cancer growth [44]. Extracellular vesicles (EV) secreted by cardiosphere cells (a heart progenitor cell) were reported to reduce HT1080 fibrosarcoma invasiveness *in vitro* and fibrosarcoma growth in a human xenograft model in nude athymic Foxn1nu mice*,* indicating that heart-derived EVs have intrinsic anti-cancer cell proliferation capacity [45]. A recent study has explored the phenotype of human stromal cells isolated from different tissues, including bone marrow, liver, adipose, and heart [46]. While stromal cells from the bone marrow, liver, or adipose tissue supported the growth of tumour cells in a co-culture setting, heart stromal cells caused a loss of viability of the tumour cells [46]. The authors noted that it was not clear from their experiments whether the growth inhibition and/or loss of cancer cell viability was caused by direct interaction of tumour cells with the stromal cells or by secretion of tumour-suppressive products.

Overall, there are few experimental studies investigating why muscles are disproportionally rare sites of primary cancers, and the precise mechanisms present a gap in our knowledge. This poses an exciting avenue for future research, as an understanding of why muscle is rarely the site of primary cancer may provide insights into environmental and genetic factors that trigger tumourigenesis.

## Incidence of striated muscle metastases

As mentioned above, the distribution of metastatic tumours is not consistent across organs, a phenomenon known as organ-specific or site-specific metastasis [60]. For example, breast cancer commonly metastasises to the lung, liver, bone, and brain [47, 48], whereas melanomas have a high affinity for the CNS [61]. While there are distinct differences in metastatic spread across tumour types, some organs are particularly vulnerable to metastases, such as the lung, and bone [47, 49, 50]. Conversely, some organs, such as the spleen and skeletal muscle are far less affected by metastatic cancer growth [7–11]. Skeletal muscle is a particularly interesting case given its substantial contribution to total adult body mass [5], and high circulatory demands during activity [62]. Reported rates of skeletal muscle metastasis range from 0.03 to 1.8% [7–11]. Contrary to these reports, one study reported the rate of muscle metastasis to be 17.5% [63]; however, it is important to note that this paper included only two cases where the cancer was considered to have embedded between the muscle fibres, resulting in a 1.0% incidence, with most cases of metastases instead embedded in the fascia.

Berge and Sievers reported the rate of metastasis to cardiac muscle to be 2–5% in patients with metastatic disease [64]. Importantly, a post-mortem examination of patients with metastatic disease found that 9.1% of patients had evidence of cardiac metastases; however, only 31.8% of these cases had metastases in the myocardium (i.e. 2.89% of total cases) [65]. Furthermore, an autopsy study found that secondary tumours of the heart occurred at an incidence of 1.23% [12]. The rarity of cardiac muscle metastases represents a particularly interesting example given the extensive perfusion of coronary vasculature and drainage of the cardiac lymphatic network. Overall, skeletal and cardiac muscle are interesting examples of organs that are rarely affected by metastasis relative to their proportion of total body composition, and high blood perfusion volumes. The mechanisms by which these organs are resistant to metastasis remain controversial.

To investigate the distribution and primary site of rare, striated muscle metastases, we completed a retrospective literature search of the PubMed database using the keyword search “muscle” or “muscular” and “metastatic” or “metastasis” or “metastases” and not “muscle mass” or “muscle loss”. Between 2010 and 2023 this search yielded a total of 469 papers (Supplemental Figure 1). Papers were excluded if they were not related (173), not in English (19), non-human (4), case-series, reviews, or commentaries (49), primary research articles (4) or did not specify the muscles affected (19), leaving 201 papers and 210 total case studies (Supplemental Figure 1). Each case study is summarised in Supplemental Table 1. Of these case studies, 114 were male, 92 were female (4 not specified), with an average age of 58.1 ± 14.5 (mean ± SD) years (Supplemental Table 1).

The most common cancer types resulting in striated muscle metastases were lung (16.2%), gastrointestinal/rectal (16.2%), breast (11.9%), kidney (7.1%), and bladder (6.7%), followed by thyroid (6.2%), cervix (6.2%), and liver (5.7%) (Table 1). These cancers comprise a large proportion of cancer cases worldwide, with breast and lung cancers being the two most common, followed by colorectal (10.7%), prostate (7.8%), stomach (6.0%), liver (5.0%), cervix (3.3%), oesophageal (3.3%), thyroid (3.2%), and bladder (3.2%) [66]. Our findings are consistent with a review of 49 studies, where the most common cancer of origin for skeletal muscle metastasis was lung followed by renal cell carcinoma [67]. Another review of 29 papers reported that lung cancers accounted for most muscle metastasis cases, followed by urological, gastrointestinal, and unknown primary tumours [68]. In contrast, a review of a hospital database, that contained 31 cases of muscle metastasis, found melanoma, unknown primary, colorectal and lung were the most common cancers of origin, in decreasing order of incidence [69]. Another review of a hospital database found similar results to our literature search, with the most common cancers of origin being the lung, breast, rectum, bladder and pancreas [70].

**Table 1:** Primary cancers of origin of muscle metastasis cases reported in the literature. Summary of clinical incidence of cancers reported in striated muscle according to site of primary cancer. See Supplementary Materials for original literature

Primary site of origin	Frequency in literature	Percent of total cases (%)
Lung	34	16.2
Gastrointestinal	34	16.2
Breast	25	11.9
Kidney	15	7.1
Bladder	14	6.7
Cervix	13	6.2
Thyroid	13	6.2
Liver	12	5.7
Pancreas	9	4.3
Skin	7	3.3
Other	34	16.9

**Fig. 1:**
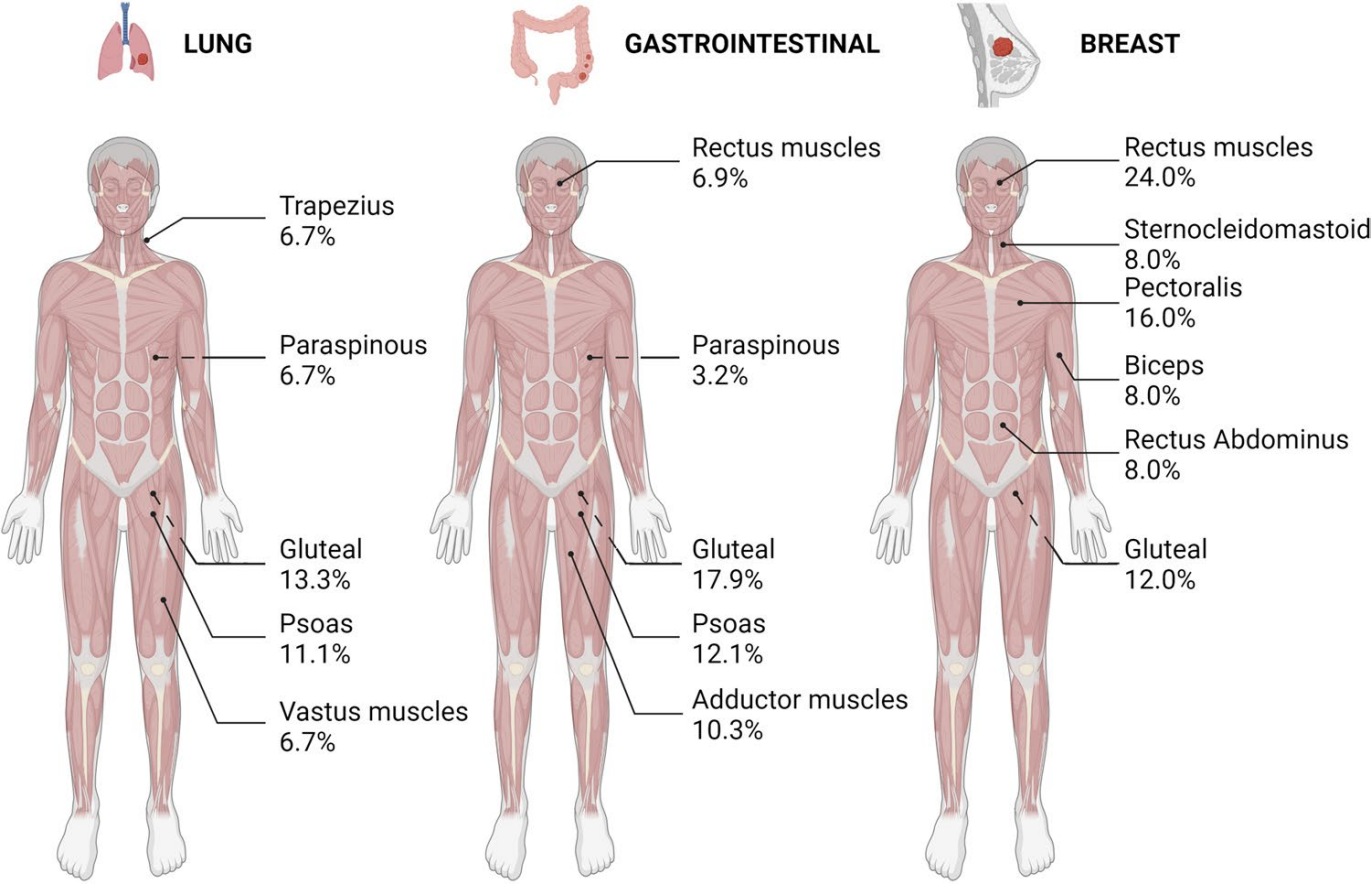
Summary of most common sites of muscle metastasis in the 3 most common muscle metastasizing primary cancers. Anatomical distribution of clinical cases of metastases identified in skeletal muscles for cancers originating in the lung, the gastrointestinal organs, and the breast. Dashes lines indicate muscle is located on posterior compartment

Over half of the muscle metastases reported occurred in limb muscles (Table 2). The most common muscles affected were gluteal muscles (12.3%), followed by psoas (10.0%), orbital rectus muscles (5.0%) paraspinous (4.7%), and deltoid (4.2%) (Table 2; Figure 1). Similar to our findings, a review of 29 papers found the psoas (13.5%), gluteal (10.4%), extraocular (5.4%), erector spinae (4.9%), and myocardial (3.0%) muscles to be the most common sites of muscular metastasis [68]. Correspondingly, a review of PET/CT imaging cases in a hospital setting revealed gluteal (15.0%), psoas (8.7%), erector spinae (8.7%), rectus abdominus (7.6%), and the latissimus dorsi (6.5%) to be the most common sites of muscle metastasis [70]. Orbital muscles provide an interesting case in comparison to other appendicular and axial muscles, given their comparatively small size, and their patterns of activity and loading in controlling eye positioning. It is also noteworthy that ocular muscles exhibit different myogenic programming and are derived from both prechordal and paraxial head mesoderm, whereas the majority of torso and limb musculature originates from the dermomyotome. Limb muscle myogenesis is dependent on Pax3 and Lbx1, whereas extraocular and facial muscles possess a unique developmental program dependent on Pixt2 [71]. Therefore, given the potential over-representation of orbital muscles as a site of metastases, whether the differing developmental origins and functional role of orbital muscles might make them more susceptible to metastasis should be explored further. Distal muscles of the upper and lower limbs (Table 2) are seemingly more rare sites of muscle metastasis. For instance, the muscles of the forearm (1.2%) and hand (1.4%) comprise less than 3% of cases, while the large muscles of the calf account for 2.6% of muscle metastases. The relatively low incidence of metastases in these extremity muscles may be due in part to more exposure of disseminating tumour cells (DTCs) to shear stress on the longer journey to these muscles, in addition to the inhospitable soil encountered within these muscles.

**Table 2:** Summary from published literature of the distribution of muscle metastases. Overview of clinical incidence of cancers reported in striated muscle according to muscle location, and muscle type. See Supplementary Materials for original literature

Region	Organ	Muscle	Frequency	Percentage of cases (%)
Head/Neck (11.70%)	Orbital (7.24%)	Rectus muscles	18	5.01%
		Oblique muscles	4	1.11%
		Not-specified	4	1.11%
	Head (2.51%)	Temporalis	5	1.39%
		Pterygoid	1	0.28%
		Masseter	3	0.83%
	Neck (1.95%)	Anterior strap	1	0.28%
		Scalene	1	0.28%
		Splenius cervicis	1	0.28%
		Sternocleidomastoid	3	0.84%
		Not-specified	1	0.28%
Chest/shoulder (12.26%)	Chest (1.95%)	Pectoralis	6	1.67%
		Serratus anterior	1	0.28%
	Shoulder (10.31%)	Deltoid	15	4.18%
		Infraspinatus	9	2.51%
		Supraspinatus	5	1.39%
		Subscapularis	6	1.67%
		Teres major/minor	2	0.56%
Back (13.09%)	Intrinsic (8.64%)	Paraspinous	17	4.74%
		Erector spinae	7	1.95%
		Quartus lumborum	4	1.11%
		Rhomboid	1	0.28%
		Levator scapulae	1	0.28%
		Longissimus	1	0.28%
	Extrinsic (4.46%)	Latissimus dorsi	5	1.39%
		Trapezius	11	3.06%
Abdominal (5.01%)		Rectus abdominus	7	1.95%
		Oblique	5	1.39%
		Abdominal wall	1	0.28%
		Intercostal	2	0.56%
		Transverse abdominus	2	0.56%
Limbs (56.54%)	Upper limb (8.36%)	Biceps brachii	10	2.79%
		Triceps	8	2.23%
		Brachialis	1	0.28%
		Forearm	6	1.67%
		Brachioradialis	2	0.56%
		Hand muscles	3	0.84%
	Lower limb (48.19%)	Gluteal muscles	44	12.26%
		Psoas	36	10.03%
		Iliacus	10	2.79%
		Piriformis	8	2.23%
		Quadratus femoris	2	0.58%
		Obturator	8	2.79%
		Pectineus	1	0.28%
		Rectus femoris	10	2.79%
		Vastus muscles	11	3.06%
		Sartorius	3	0.84%
		Hamstring muscles	12	3.34%
		Adductor muscles	18	5.01%
		Gastrocnemius	5	1.39%
		Soleus	3	0.84%
		Peroneal muscle	1	0.28%
		Tibialis anterior	1	0.28%
Cardiac (1.39%)			5	1.39%

## Proposed mechanisms to protect striated muscle from metastasis

**Fig. 2:**
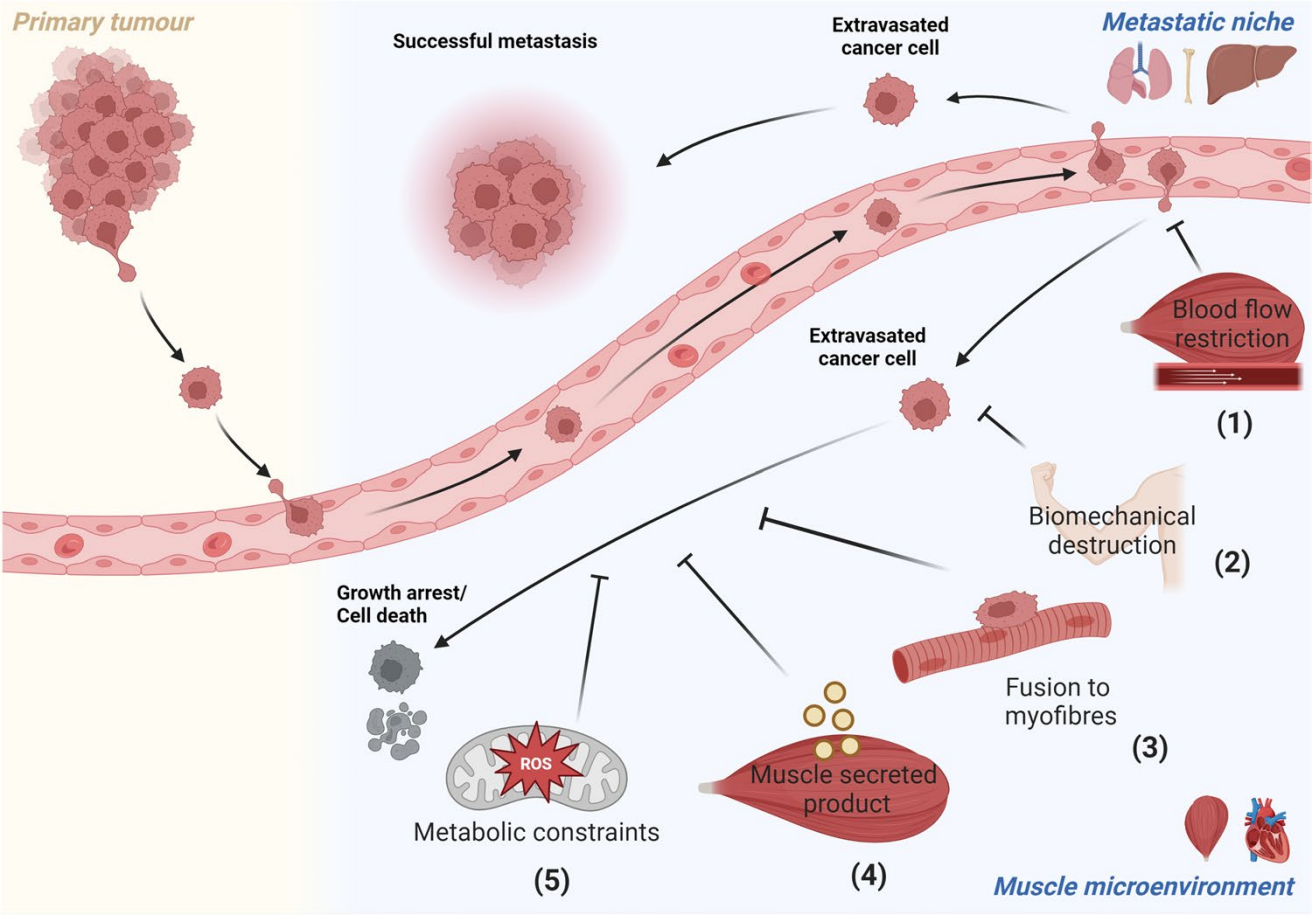
Proposed mechanisms to explain why muscle is resistant to metastasis. The current state of the literature proposes that either one or a combination of (1) blood flow restriction, (2) biomechanical destruction, (3) fusion to muscle fibres, (4) a secreted muscle product, or (5) redox balance preventing successful metastatic colonisation in muscles. Tumour cells are depicted in pink, dormant/apoptotic cells are depicted in grey

To date, several hypotheses have been proposed in an attempt to explain why skeletal muscle is resistant to metastasis (Fig. 2). Below we provide an overview of the different hypotheses with the evidence for and against the concept and limitations in their interpretation.

### Does blood flow to muscle prevent metastatic seeding?

**Fig. 3:**
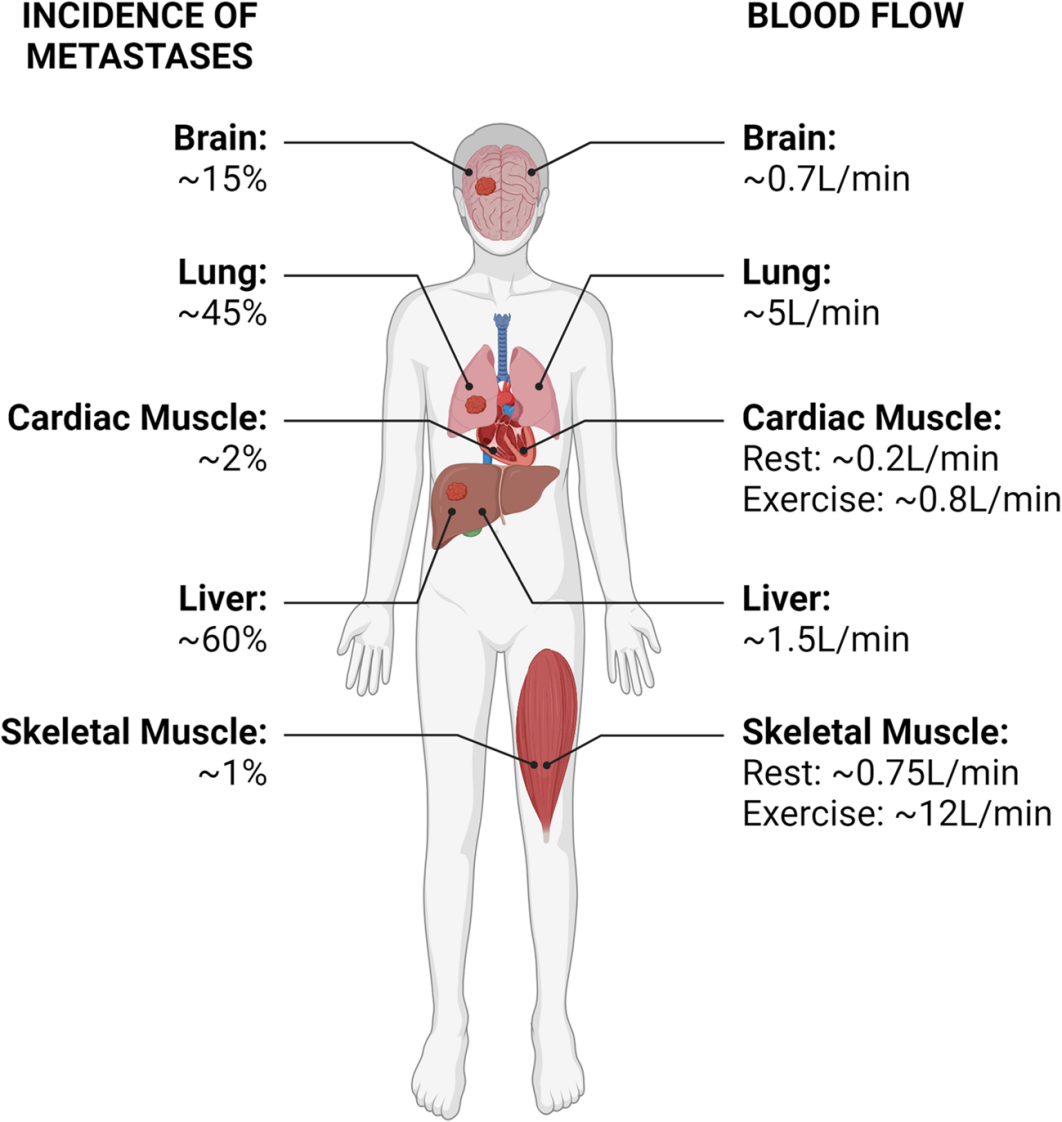
Does blood flow to muscle prevent metastatic seeding? Based on the vascular flow theory of metastasis, does the flow of blood to muscle prevent seeding of metastatic cells in muscle? Blood flow rates to muscle are comparable to some metastasis prone organs. Muscle undergoes rapid increases in blood flow during exercise which could deter metastatic seeding. Rates of metastasis collated from [7–11, 47–50], blood flow rates to different tissues summarised from [51–54]

It has been proposed that blood flow restricts the opportunity for circulating tumour cells to colonise muscle (Fig. 3). As mentioned earlier, James Ewing suggested that blood flow and haemodynamics dictate the spread of metastases [3]. A recent study found that circulating tumour cells (CTCs) have a higher metastatic potential and intravasate at higher rates during sleep, suggesting that physiological parameters that vary markedly over the course of daily activity patterns, which include marked alterations in blood flow to specific tissue beds - may influence metastatic seeding [72]. Organs such as the liver, lung, and bone have rich blood supplies but exhibit much higher incidences of metastasis than striated muscles [17] (Fig. 3). What potentially differentiates skeletal muscle is that its local blood flow can change rapidly when it is recruited for contraction (Fig. 3). These rapid changes in blood flow may prevent deposition of circulating tumour cells and thus limit their ability to extravasate. However, extended periods of relative inactivity, such as during sleep, or in physically inactive patients confined to extended bedrest would create scenarios in which muscles exhibit comparatively low perfusion rates, which might conceivably be more conducive for cancer cell lodgement within muscles. Studies entailing inoculation of colon cancer cells directly into the circulation of rats *via* intra-arterial injection reported that the lung and liver contained high numbers of cancer cells after 30 min, whereas the abdominal rectus muscle contained fewer cells [73]. Studying the flow of tumour cells in the microvasculature of mice with high-resolution intravital video-microscopy found that B16-F10 melanoma and D2A1 mammary tumour cells arrest in the cremaster muscle after just 1 minute in the circulation [55]. Furthermore, Crist and colleagues have shown recently that DTCs are able to traffic to and persist within human skeletal muscle from patients who had died from metastatic breast cancer [22]. Also, they showed that muscle tissue from mice inoculated with MDA-MB-231 breast cancer cells into their mammary fat pad contained tumour cells. These studies suggest that tumour cells can still circulate to, and arrest in, skeletal muscles. Similar observations have also been made in cardiac muscle where evidence of microscopic metastases has been reported in 4T1 tumour-bearing mice [74]. Thus, while there may be some haemodynamic limitations that reduce the potential for muscle metastatic colonisation, it is likely that there are other factors within striated muscles that deter overt metastases from occurring.

### Does biomechanical destruction prevent cancer growth?

**Fig. 4:**
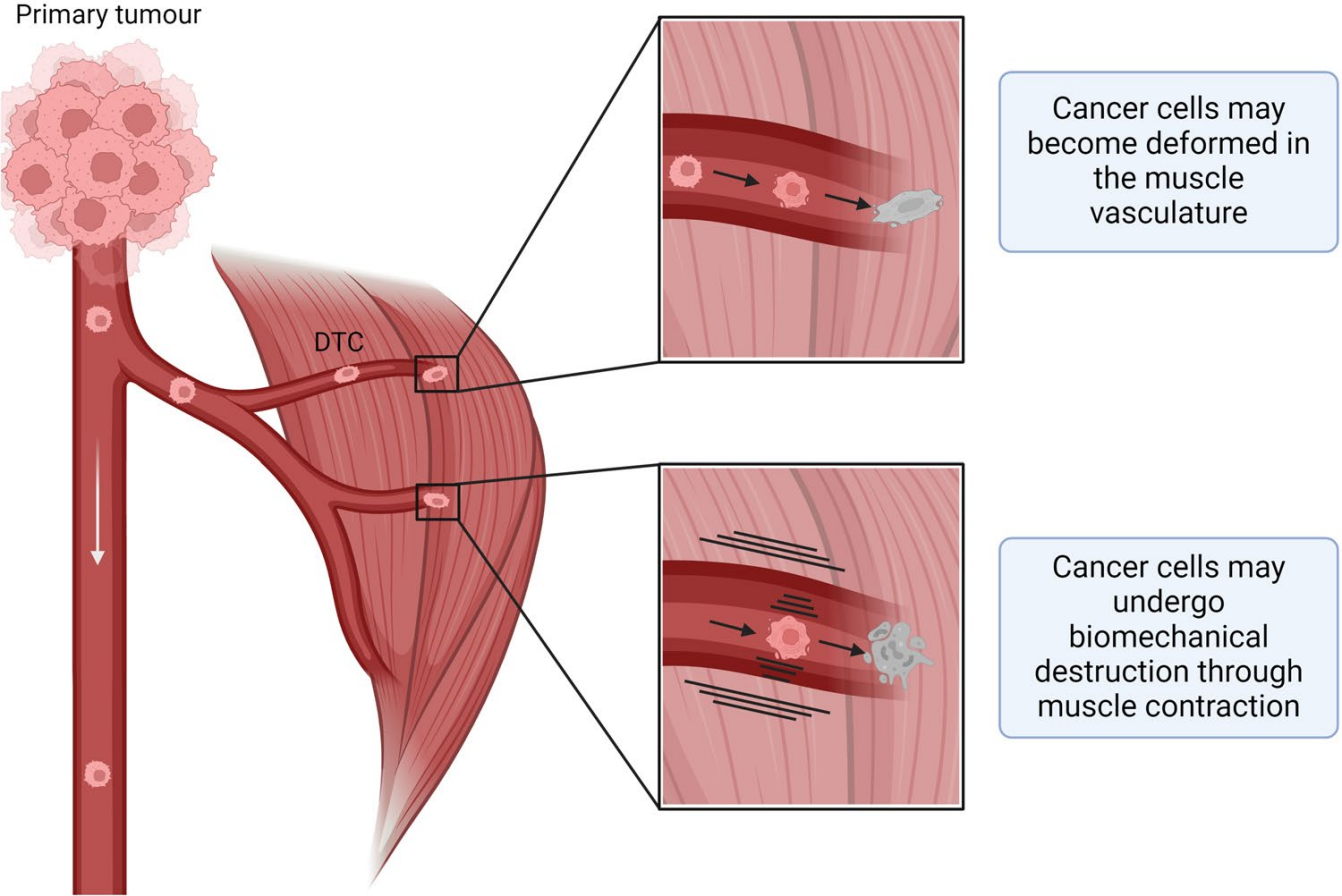
Does biomechanical destruction prevent cancer growth? Based on studies by Morris et al. [55] and Hayashi et al. [56], there is evidence that cancer cells become deformed as they enter muscle. Evidence from Weiss [18, 57] suggests that biomechanical forces within muscle can deter muscle metastasis. Disseminating tumour cells (DTC) are depicted in pink, deformed cancer cells are depicted in grey, blood vessels are shown in dark red

Biomechanical destruction of cancer cells in skeletal muscle is another proposed mechanism behind the protection of muscle from metastasis (Fig. 4). This hypothesis was tested by Weiss in 1989, who denervated the femoral nerve in the hindlimbs of mice that also had B16 murine melanoma cells injected into their left cardiac ventricle, which resulted in an increased incidence of tumour formation in the quadriceps femoris muscle following histological observation [18]. Interestingly, electrical stimulation of denervated hindlimb muscles largely reduced tumour formation to that of control muscle or those with stimulation alone. Weiss, therefore, proposed that the inactivity of the denervated muscle was the cause of increased tumour colonisation and growth. Moreover, from these observations Weiss proposed that muscle motion could destroy circulating tumour cells that enter muscles, thereby inhibiting extravasation into muscle and metastatic colonisation [18]. This evidence is supported by a study in which HT-1080 fibrosarcoma cells that were residing in skeletal muscles following inoculation into the tail veins of nude mice, were highly deformed after 48 h, compared to cancer cells in lung and liver, as measured by analysis of cytoplasm and nuclear dimensions [56]. Similar observations have been reported with murine melanoma and breast cancer cells that were more deformed in the mouse cremaster muscle than in the liver [55]. These results indicate that the muscle environment may mechanically destroy cancer cells [56]. Weiss proposed that a similar mechanism is applicable to the heart, where a majority of cells administered to mice via an intra-cardiac injection were destroyed within 5 min [57]. A limitation of these studies, however, was the experimental model whereby cancer cells were disseminated following intracardiac injection. As such, these cells do not exhibit a spontaneously metastatic phenotype and have not been exposed to modifying factors in the primary tumour. Furthermore, these studies do not elucidate whether biomechanical destruction occurred, or if muscle motion led to the production of secreted factors within the muscle environment that inhibited metastasis formation in muscle. Given that a degree of physical movement is evident in other tissues where metastases occur at higher frequencies (such as the lung, and regions of the GI tract), and in the absence of other reports indicating that muscle is protected from metastasis due to biomechanical forces, it would appear other mechanisms may be more significant in explaining the comparative resilience of striated muscle to colonisation by metastatic cancers.

### Are metastases inhibited due to fusion of cancer cells with muscle fibres?

Decades ago, Seely hypothesised that the environment within a muscle may either be hostile to tumour cells, or that tumour growth was inhibited by elements of the muscle environment [75]. Clinical support for this concept included observations that metastases have been detected at sites of previously documented muscle trauma in humans [76], indicating that a disruption of the muscle environment may diminish muscle anti-cancer activity. Although the clinical findings are interesting, no mechanism was tested in the published reports. As potential mechanisms for their observations, the authors suggested that injury of muscle might support metastasis through altered muscle physiology, such as contractile activity, pH changes, temperature, or blood supply [76].

**Fig. 5:**
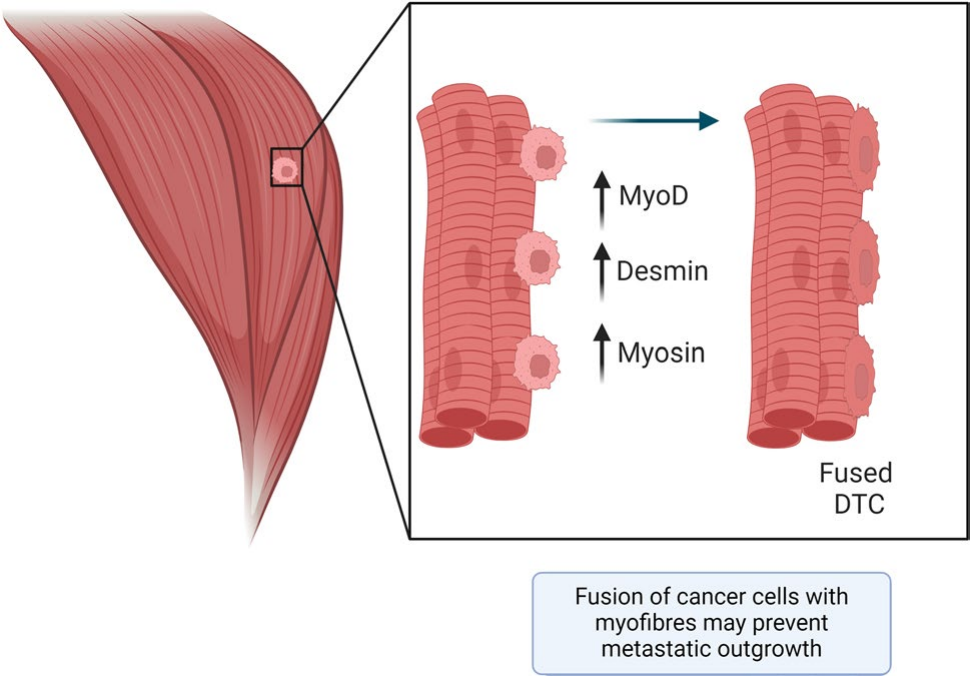
Are metastases inhibited due to fusion of cancer cells with muscle fibres? Parlakian et al. [19] found that tumours directly injected into muscle fused with muscle fibres. These fused cells expressed myogenic transcription factors MyoD, Desmin, and Myosin. Cancer cells are depicted in pink, muscle fibres are represented in red, cancer cells fused to muscle fibres are depicted in red

As an alternative mechanism, Parlakian and colleagues reported that the inhibition of metastasis in muscle may be caused by direct fusion of cancer cells with muscle fibres (Fig. 5). To investigate the concept that the muscle environment inhibits metastatic growth, Parlakian and colleagues found that following intravenous and intraperitoneal injection of B16 melanoma cells into C57Bl/6 mice, metastases formed in several organs, including the lung, liver, intestine, bone, and heart, but never in skeletal muscle [19]. Of the cells found in heart, across two independent experiments, only one in three or one in four metastases occurred in the myocardium [19]. These observations may point to differences in the resistance of cardiac and skeletal muscles to tumour cell colonisation, as skeletal muscle fibres may be more likely to fuse with cancer cells than cardiac muscle fibres. Parlakian and colleagues did not follow up on this particular result, which poses an area for future research. In subsequent experiments, where B16 cells were injected directly into skeletal muscles, the team observed small melanoma nodules that displayed well-defined boundaries from the skeletal muscle cells [19]. These observations suggested that the muscle environment was preventing metastatic outgrowth. Furthermore, through the use of a co-culture model, Parlakian and colleagues observed that the myogenic markers, Desmin, Myosin, and MyoD, were elevated in B16 melanoma and Lewis lung cancer cells when cultured with primary human myoblasts [19]. It was not investigated whether the elevated abundance of Desmin, Myosin, and MyoD was caused by fusion with muscle cells or was due to the transfer of these factors from muscle-derived micro-vesicles. In another report, co-culture of immortalised myognic cells of the mouse C2C12 line was shown to prevent 4T1 breast cancer cell proliferation *in vitro* [77]. Downregulation of the myogenic program transcription factor, MyoD in C2C12 cells, reversed the growth suppression of 4T1 cells [77]. Unfortunately, the team did not follow up to determine which genes within the myogenic transcriptional program contributed to the growth suppressive effects on the 4T1 cells. Overall, these data indicate that transcriptional programs and/or factors present within muscle cells and muscle fibres, rather than blood flow restrictions or biomechanical forces, may play a role in preventing muscle metastasis.

### Do striated muscle cells secrete a product with tumour suppressive activity?

**Fig. 6:**
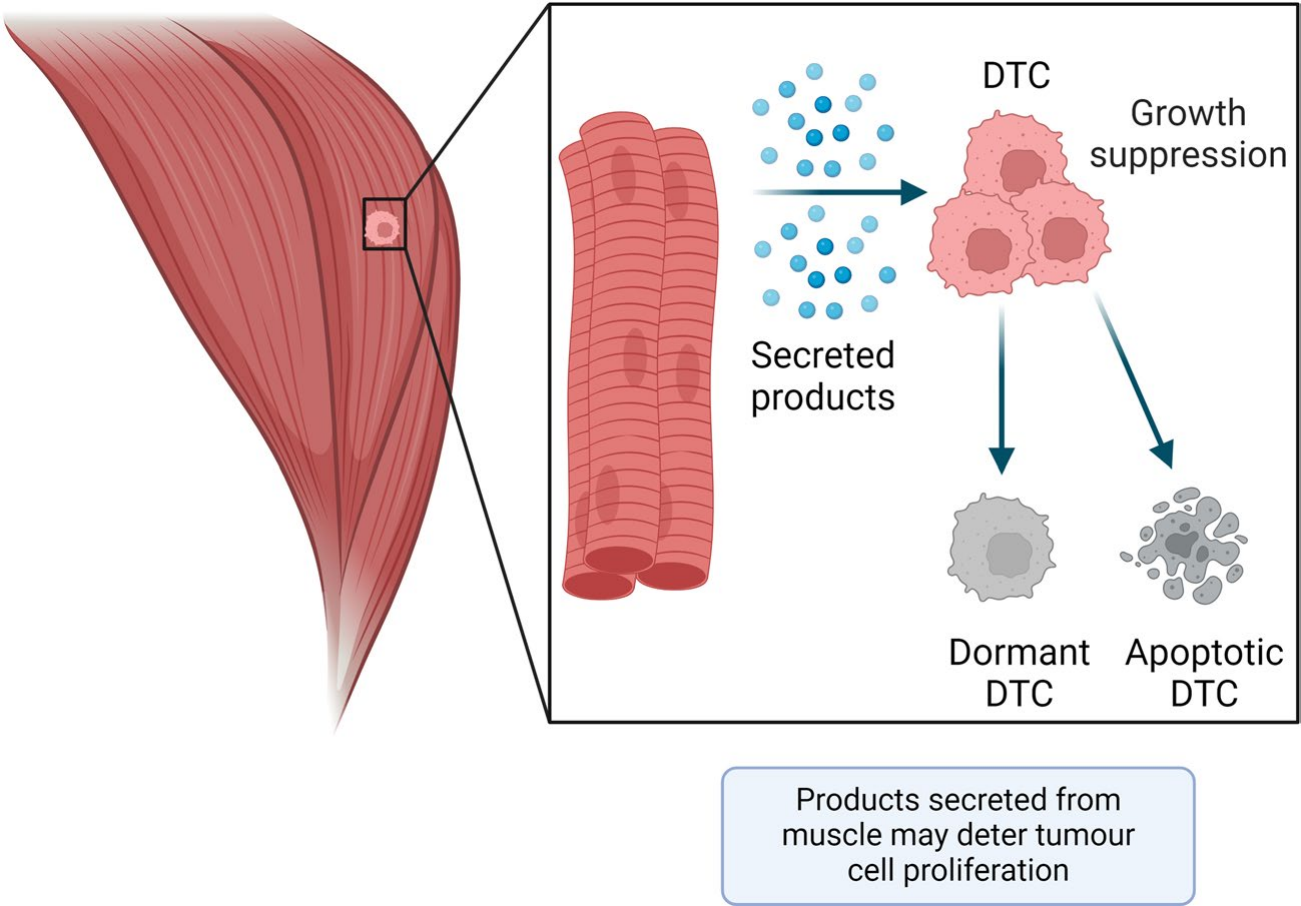
Do striated muscle cells secrete a product with tumour suppressive activity? Experimental data from Djaldetti et al. [20], Luo et al. [21], Fishman et al. [58], and Bar-Yehuda et al. [59] suggest that a secreted product produced by muscle can deter cancer cell growth and metastasis. The precise mechanism behind this growth suppression, and the specific metastasis suppressive products remain elusive. Disseminating tumour cells (DTC) are depicted in pink, dormant/apoptotic DTCs are shown in grey, and muscle fibres represented in red

Several research groups have explored the concept that striated muscle cells may deter metastasis by secreting factors that prevent the growth of cancer cells (Fig. 6). For instance, conditioned medium from myogenic cells applied to cultured cancer cells decreased the number of viable cancer cells *in vitro*, as tested for multiple cell lines [20, 21]. This activity was observed to be dose-dependent, with a reduction in cancer cell number being positively correlated with increasing concentrations of myogenic cell conditioned medium [58]. Parlakian and colleagues also found that the proportion of B16-F10 melanoma cells exhibiting apoptotic markers relative to those that displayed proliferative signatures was increased following the addition of C2C12 conditioned medium, suggesting that the secretome of the muscle environment may sensitise cancer cells to apoptotic mechanisms [19]. Whilst these cell culture studies present findings consistent with an anti-proliferative effect of muscle-secreted factors upon cancer cells, an important limitation to consider is that the cell culture conditions employed did not study the process of metastasis. These limitations have been considered in studies from Bar-Yehuda *et al.* who investigated the effects of muscle-conditioned medium on metastasis formation *in vivo* [59]. Mice that were injected intravenously with B16-F10 melanoma cells and were subsequently administered muscle-conditioned medium four times daily for 15 days by oral delivery, exhibited fewer metastatic lesions in vulnerable organs than control mice [59]. An earlier study by the same team had suggested that adenosine in the conditioned medium was the active component mediating the anti-metastatic effect [58]. However, administration of adenosine alone did not exert any anti-metastatic activity in other organs, which indicated that cultured muscle cells likely release factors other than adenosine to contribute to the inhibition of secondary tumour growth in adult mammalian skeletal muscle [59]. Therefore, the concept that products secreted by skeletal muscles prevents metastasis is compelling and is deserving of further investigation.

Further evidence for the anti-metastatic niche has been shown in muscles of the heart. As the myocardium is rarely the site of metastases, Erin et al used this tissue as a model system to study the role of sensory neurones in metastatic colonisation, by delivering a neurotoxic dose of capsaicin to inactivate sensory nerve fibres in 4T1 tumour-bearing mice [74]. The authors showed that this manipulation resulted in more spontaneous lung and heart metastasis [74, 78]. These spontaneous heart metastases occurred in both the pericardium and in the myocardium [74, 78]. As disruption of the microenvironment within the heart resulted in spontaneous myocardial metastasis, this supports the notion of the host environment as an important regulator of metastatic colonisation. Erin et al also developed three clones of 4T1 tumours with a propensity to metastasise to the heart, liver, and brain, respectively [79]. The liver metastatic clone possessed a higher migratory rate compared to the other clones [79]. The epithelial marker E-Cadherin was increased in the brain metastatic clone, suggesting that E-Cadherin may play a role in brain metastasis. The EMT markers N-Cadherin and alpha-smooth muscle actin were also lower, whereas Vimentin expression was higher in heart metastatic cells, indicating that the properties of the tumour cell itself can dictate metastatic fate. Collectively, these observations argue for a determining role of the cancer cell “seed” as well as a “soil” that is compatible for metastatic cell outgrowth.

### Do metabolic constraints within striated muscle prevent tumour cell proliferation?

**Fig. 7:**
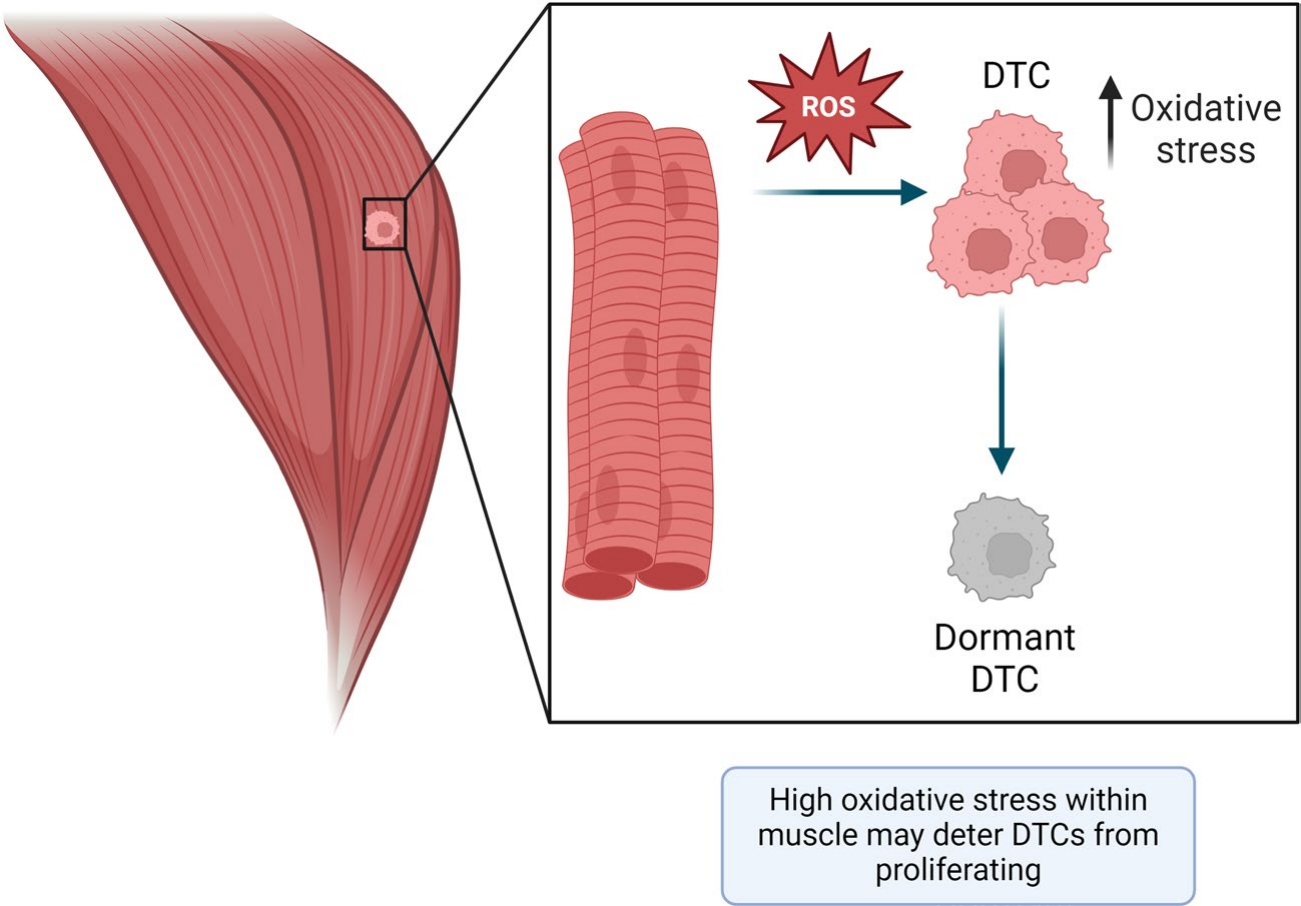
Do metabolic constraints within striated muscle prevent tumour cell proliferation? Crist et al [22] showed that the skeletal muscle microenvironment causes unchecked oxidative stress in disseminated tumour cells (DTC), which prevents these cells from proliferating. DTCs are depicted in pink, dormant DTCs are shown in grey, and muscle fibres are represented in red

Recently, metabolic constraints within skeletal muscle have been proposed as a protective mechanism [14] (Fig. 7). Through co-culture of breast cancer cells with lung fibroblasts and differentiated myotubes, Crist and colleagues showed that skeletal muscle-conditioned medium and skeletal muscle-derived EVs had no effect of tumour cell growth [22]. These findings differ from earlier studies that reported the inhibitory effects of muscle conditioned medium on tumour cell proliferation *in vitro* and tumour growth *in vivo*. Crist *et al.* proposed an alternative mechanism, whereby the redox state of muscle prevents cancer cell outgrowth [22]. The authors reported that DTCs encounter high oxidative stress compared to DTCs in the lung, and that this stress prevents metastatic cell outgrowth [22]. In support of their proposition, when human MDA-MB-231 or murine EO771 breast cancer cells expressing mitochondrial catalase (mCAT) to neutralise reactive oxidative stress (ROS) were injected directly into the hindlimb muscles of mice, 6 out of 19 treated animals examined 6 weeks later developed a tumour signal compared to 1 out of 19 mice administered with control cancer cells. When global transgenic mice expressing mCAT were injected with the mCAT-expressing tumour cells, there was no additive effect on intramuscular metastatic burden. These data indicated that intramuscular reactive oxygen species concentrations may inhibit metastasis. It is important to note, however, that whilst expression of mitochondrial CAT (mCAT) and cytosolic CAT (cCAT) resulted in increased transition out of the single cell state compared to un-modified tumour cells, these cells typically remained in small clusters and only one mouse in the cohort developed an overt muscle metastasis [22]. Furthermore, when the effect of mCAT was explored in the lung, the mice exhibited fewer metastatic lesions, in contrast to the results in skeletal muscle [22]. These results indicate that an environment that has either too high or insufficient oxidative stress may deter metastatic cell outgrowth. Therefore, whilst it is evident that metabolic constraints within the muscle have a role in protecting muscle, as Crist and colleagues noted, this mechanism deserves further examination.

### Are there other mechanisms that could deter muscle metastases?

**Fig. 8:**
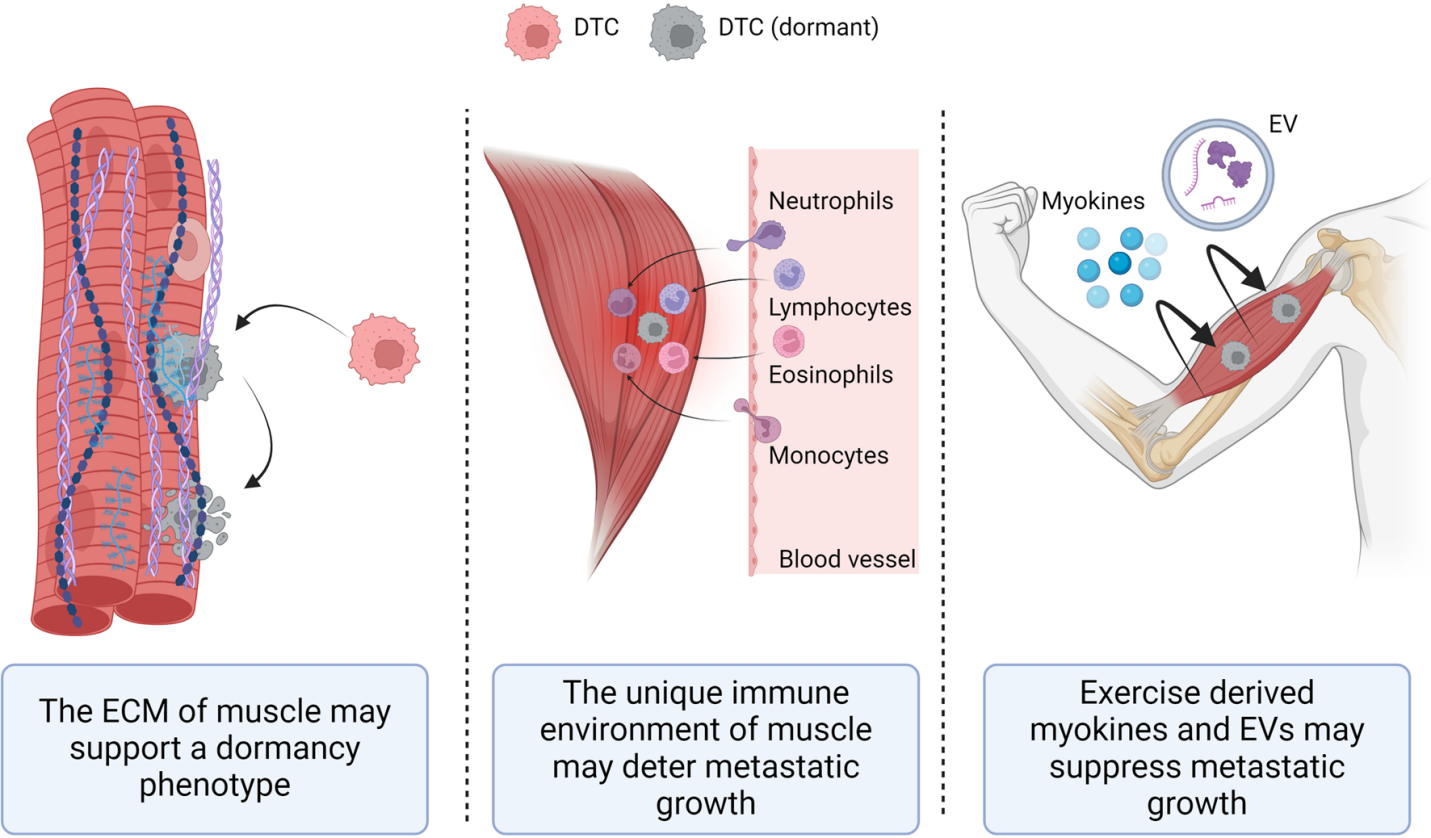
Are there other mechanisms that could deter muscle metastases? Despite numerous studies investigating why metastases rarely grow within muscle, the precise tumour-suppressive mechanisms have not been fully elucidated. There could also be alternative mechanisms at play, including the unique ECM of muscle (left), the immune microenvironment in muscle (centre), or muscle activity producing myokines or extracellular vesicles (EV) (right) suppressing muscle metastatic outgrowth. Disseminating tumour cells (DTC) are depicted in pink, dormant DTCs are shown in grey, and muscle fibres are represented in red

Presently, it cannot be ruled out that there are other mechanisms that contribute to the comparative resistance of muscle to metastasis development, that have yet to be fully recognised (Fig. 8). For example, Crist and Ghajar proposed that the composition of extracellular matrix (ECM) present in muscle may induce metastatic dormancy [14]. In skeletal muscle, collagen VI and laminins are dominant components of the ECM resident between muscle fibres [80]. Satellite cells in skeletal muscle remain quiescent in an uninjured muscle, where they remain in contact with the ECM [81]. For instance, Collagen VI reduces myogenic commitment and can promote self-renewal of satellite cells in skeletal muscle [81]. Furthermore, *in vitro* data suggest that muscle satellite cells are capable of producing ECM collagens to maintain their quiescence [82]. We previously speculated that contact inhibition with quiescent and post-mitotic cells may prevent primary tumour development in muscle. Thus, these mechanisms may be similar in the suppression of metastatic tumours. Whether the ECM in muscle may provide specific cues that induce quiescence and dormancy in DTCs warrants further investigation.

Another potential hypothesis concerns the functionality of immune cells within the muscle, as compared to other traditionally metastasis-prone tissues. The composition of the muscle immune environment has not been explored in the context of muscular metastasis. Some tissues in the body are considered immune privileged where, despite the presentation of antigens, these tissues elicit a greatly diminished immune response. There have been some suggestions that skeletal muscle may be considered a “non-classical” immunologically privileged tissue, as the extensive vascular and lymphatic networks enable perfusion and extravasation of circulating cells, yet there are comparatively fewer immune cells that typically reside within muscle relative to other tissues [83]. However, following injury of skeletal muscle, there are marked increases in immune cell populations. Initially, there is a recruitment of monocytes that exhibit an M1, pro-inflammatory phenotype [84, 85]. This is typically followed by an influx of eosinophils and neutrophils which further promote an inflammatory microenvironment [86, 87]. Lymphocyte infiltration also occurs at the site of the wound, with immunosuppressive T regulatory cells (Tregs) playing an important role in the shift from a proinflammatory to an anti-inflammatory environment [84, 88]. Some evidence suggests that skeletal muscle-resident Tregs display a distinct transcriptomic profile compared to Tregs residing in other organs [89]. As such, it is possible that skeletal muscles feature a unique immune cell environment. Further exploration of the muscle immune response to cancer could yield valuable insights into the mechanisms of cancer metastasis.

It is well established that exercise can reduce the risk of developing cancer, and also improve survival for patients who have already been diagnosed with cancer [90]. As previously discussed in this review, muscle motion has been shown to play a role in protecting muscle from metastasis [18, 57]. However, exercise effects may not be limited to just motion – as exercise can lead to the secretion of myokines [91]. Hojman and colleagues demonstrated that serum from mice that underwent exercise, or media from electrically stimulated myotubes, inhibited MCF-7 breast cancer cell proliferation [92]. These results have also been replicated in an experimental metastasis model, where 4T1 murine breast cancer cells were administered via tail vain injection in Balb/c mice, and metastatic tumour formation was reduced with either low or moderate exercise [93]. Exercise also results in the production of muscle-derived EVs which have been reported to suppress metastasis in other organs [94]. Whether muscle-derived EVs play a role in metastasis suppression in the local muscle environment could be further explored. These exercise-derived factors could also play a role in protecting muscle from metastasis. Seely also proposed that lactic acid production in skeletal muscle may deter metastatic tumour development [75]. Whilst this mechanism has not been explored experimentally, it does suggest a role for exercise in suppressing muscle metastasis. In this review, we investigated which muscles are most affected in clinical case studies of rare muscle metastasis, and found that extraocular muscles, which do not play a large role in exercise were one of the muscles more commonly affected. However, muscles such as the deltoid and trapezius, and several lower limb muscles, which play a large role in locomotion and exercise, are also frequently affected. Consequently, there exist opportunities to learn more about the potential role of exercise derived myokines and muscle-derived EVs as potential suppressors of metastasis in muscle and more widely in other tissues.

## Conclusions and future directions

In exploring the comparative resistance of striated muscle to primary and secondary cancer, others have presented several mechanisms to explain why muscle is resistant to cancer growth. As summarised herein, there is limited evidence to indicate that blood flow to muscle prevents successful extravasation of circulating tumour cells to muscle, and recent evidence would suggest that tumour cells are able to successfully traffic to skeletal [22] and cardiac muscles [74]. Moreover, the extensive perfusion of cardiac muscle does not support the concept of inadequate access of tumour cells to striated muscle. The hypothesis that biomechanical destruction prevents colonisation of muscle needs further investigation. It is possible that the results produced by Weiss in mouse models of denervated limb muscles [18, 57] do not show that muscle contraction destroys cancer cells, but rather that the secondary effects of muscle inactivity and subsequent changes in the muscle microenvironment (including changes in ECM composition and the activity of non-muscle cell populations) promote an environment that is more supportive of tumour growth. Thus, the long-standing hypotheses that the unique blood flow and contractile properties of muscle make it resistant to metastatic growth lack sufficient evidence to be conclusively considered as the key protective mechanisms.

Several recent publications have suggested that the muscle microenvironment is responsible for the infrequent colonisation of secondary tumours in muscle. The metabolic environment has been postulated to prevent tumour growth in muscle [17, 76], with recent evidence indicating that high ROS levels in muscle prevent metastatic outgrowth [22]. However, it has also been observed that experimentally increasing the degree of oxidative stress in other traditionally metastasis-prone tissues can also increase cancer colonisation, which illustrates the complexity of this mechanism. Alternatively, secreted products from muscle may prevent the growth of muscle metastases. Thus, the muscle environment might be suppressing cancer growth by inducing a dormancy phenotype in the extravasated cancer cells. Whilst there is supportive evidence indicating that conditioned medium from muscle cells can prevent tumour growth, identification of the factors that muscle may be secreting to deter growth has remained elusive.

An understanding of the environmental cues in tissues that allow for tumourigenesis and growth of cancers is an area of intense interest in cancer research. Determining the mechanisms that make an environment resistant to hosting cancers is an exciting prospect, as the findings may provide insights into strategies that could help to prevent cancer cell growth in traditionally vulnerable tissues. Given the significance of metastasis as a contributor to many cancer-related deaths, better defining the factors that make a specific tissue microenvironment unfavourable for cancer cell colonisation and subsequent growth is particularly compelling.

It is important to note that not all mechanisms presented in this review may be equally amenable to the development of novel anti-metastatic therapies. For instance, replicating the unique blood flow dynamics of muscle in other organs would be an unlikely therapeutic strategy. Equally, biomechanical forces, and fusion to muscle fibres would be difficult to translate to other organs. However, identification of putative muscle-derived secreted products could be leveraged as potential anti-metastatic therapies. Bar-Yehuda et al administered conditioned media from muscle cells to mice and observed reduced metastasis [59]. Therefore, if the specific anti-metastatic products secreted from muscle could be identified, isolation of these factors could offer a potential novel therapeutic strategy. Crist and colleagues demonstrated that altering the redox state in tissues can influence cancer cell fates, but were unable to demonstrate that manipulation via this approach could reduce metastasis in traditionally vulnerable tissues [22]. As such, further study is needed to determine if altering the redox state of at-risk organs could be a feasible therapeutic strategy.

Recent advances in technologies and research capabilities may allow deeper investigation of some of these unanswered questions. The emergence of single-cell sequencing could be leveraged to understand the transcriptomic signal of a DTCs in muscle. Proteomic analysis of these tumour cells may also reveal novel tumour-suppressive mechanisms. Conceivably, in-depth examination of cancer cell responses following exposure to products derived from muscle may prove instructive in identifying signalling mechanisms and programs of transcription that contribute to inhibition of cancer cell proliferation. Recovery of rare muscle-disseminated tumours may also prove useful, though the scarcity of such cells presents its own challenges as far as identifying early mechanisms that enable initial metastatic establishment. Utilisation of spatial transcriptomic techniques [95] could also be an effective means by which to explore the interactions of cancer cells and their host microenvironment.

The observations reported to date indicate that there are legitimate reasons to consider striated muscle comparatively resistant to primary and secondary cancers, and that there are mechanisms still to be fully understood, but deserving of further investigation. Given the intriguing results of the aforementioned pre-clinical studies reported to date, it is exciting to contemplate what deeper investigation of striated muscle might reveal about the biology of cancer, and the prospects for developing new strategies to reduce the impact of metastatic disease.

## Supplementary information

ESM 1 Supplemental Fig. 1: Literature search strategy. Supplemental Table 1: Case studies identified and analyzed to compile Tables 1 and 2. (DOCX 393 kb)

## Data Availability

Analyses of published literature describing metastasis are provided in the manuscript and Supplemental Information. Relevant calculations are available on request.
