# Supplementary material for: Striated muscle: an inadequate soil for cancers
Source: Cancer Metastasis Rev. 2024 Jul 12;43(4):1511–27. doi: 10.1007/s10555-024-10199-2 (PMC11554797; doi:10.1007/s10555-024-10199-2)
Supplement: Supplementary file 1 — Supplemental Fig. 1: Literature search strategy. Supplemental Table 1: Case studies identified and analyzed to compile Tables 1 and 2. (DOCX 393 kb) [file 10555_2024_10199_MOESM1_ESM.docx]

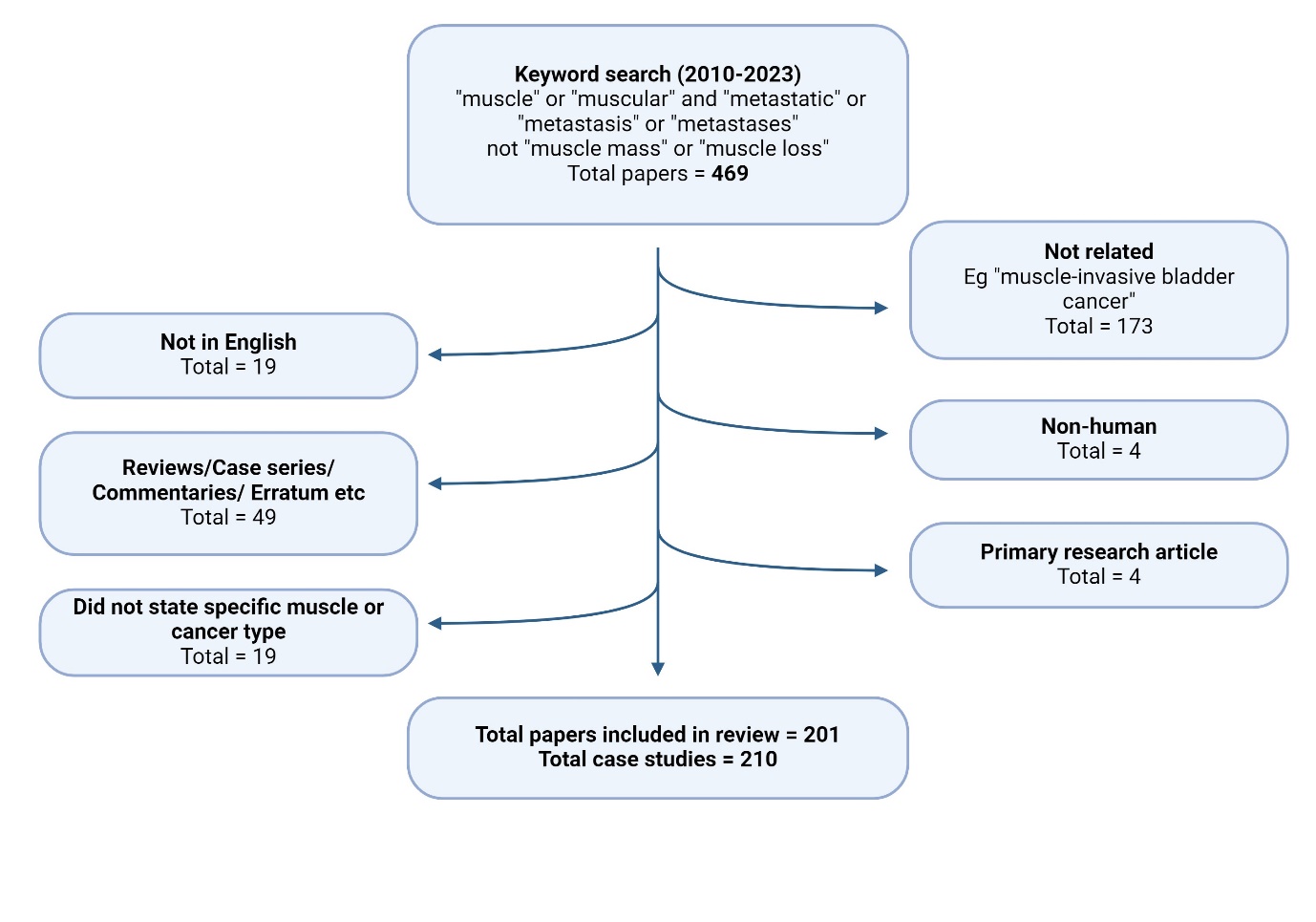


**Supplemental Figure 1: Literature search strategy.**

**Supplemental Table 1: Case studies identified and analyzed to compile Tables 1 and 2.**

| **Author** | **Primary cancer** | **Muscle site(s)** | **Sex** | **Age** |
| --- | --- | --- | --- | --- |
| [1] | Mixed germ cell ovarian cancer | L. infraspinatus, paraspinal, L. rectus femoris, sartorius posterior knee flexor | Female | 25 |
| [2] | Lung squamous cell carcinoma | R. brachioradialis | Male | 65 |
| [3] | Gastric adenocarcinoma | R. quadratus lumborum, gluteus maximus, L. vastus lateralis | Female | 57 |
| [4] | Hepatocellular carcinoma | Semimembranosus, semitendinosus | Male | 55 |
| [5] | Cervical squamous carcinoma | Psoas | Not specified | 64 |
| [6] | Ductal carcinoma | L. Biceps | Female | 60 |
| [7] | Nasopharyngeal Squamous Cell Carcinoma | L. psoas | Male | 50 |
| [8] | Melanoma of unknown primary | Gluteus maximus | Male | 43 |
| [9] | Lobular breast carcinoma | Anterior strap | Female | 71 |
| [9] | Breast adenocarcinoma | L. latissimus dorsi | Female | 73 |
| [10] | Melanoma | L. lateral rectus | Female | 80 |
| [11] | Gastroesophageal junction adenocarcinoma | R. psoas | Male | 65 |
| [12] | Papillary thyroid cancer | R. vastus medialis | Female | 31 |
| [13] | Cholangiocarcinoma | L. psoas | Female | 34 |
| [14] | Pulmonary adenocarcinoma | L. paraspinal | Female | 62 |
| [15] | Gastrointestinal stromal tumor | Cardiac | Male | 56 |
| [16] | Cervical carcinoma | R. psoas | Female | 52 |
| [17] | Pancreatic cancer | Paravertebral, obturator, gluteal | Female | 58 |
| [18] | Infiltrating ductal carcinoma | R. pectoralis major, R. gluteal | Female | 56 |
| [19] | Colorectal adenocarcinoma | L. medial/ posterior thigh, R./L. gluteal | Male | 36 |
| [20] | Oesophageal adenocarcinoma | Medial rectus | Male | 51 |
| [21] | Renal Cell Carcinoma | R. deltoid | Male | 69 |
| [22] | Renal cell carcinoma | L. psoas | Male | 63 |
| [23] | Papillary carcinoma of the thyroid | R. adductor long, R. iliopsoas | Female | 68 |
| [24] | Bladder cancer | L. obturator internus | Male | 83 |
| [25] | Duodenal leiomyosarcoma | R. Psoas | Female | 61 |
| [26] | Sacral chordoma | L. trapezius, R. paraspinal, infraspinatus, L. bicep, R. triceps, R. gluteal, L. vastus intermedius | Male | 65 |
| [27] | Lung carcinoma | L. triceps | Male | 61 |
| [28] | Neuroendocrine breast tumor | R. biceps brachii | Female | 69 |
| [29] | Papillary thyroid carcinoma | L. trapezius, R. subscapularis | Female | 50 |
| [30] | Bronchopulmonary carcinoma | R. lateral pterygoid | Not-specified | 70 |
| [31] | Pancreatic cancer | Peroneal, quadriceps, deltoid | Male | 55 |
| [32] | Breast carcinoma | R. brachioradialis | Female | 76 |
| [33] | Gastrointestinal stromal tumor | L. adductor longus | Female | 23 |
| [34] | Breast cancer | Pectoralis | Female | 27 |
| [35] | Dedifferentiated liposarcoma or heart sarcoma | L./R. adductor magnus, L. subscapularis, R. longissimus, R. gluteus medius, L. pectinate (either heart metastasis or primary) | Female | 59 |
| [36] | Renal cell carcinoma | Rectus femoris | Female | 75 |
| [37] | Melanoma of unknown primary | R. temporalis | Female | 85 |
| [38] | Non-Small Cell Lung Carcinoma | R. Iliac | Male | 65 |
| [39] | Gastric adenocarcinoma | L. supraspinatus, subscapularis, R./L. biceps brachii, paraspinal, R. gluteus maximus, L. psoas | Male | 42 |
| [40] | Orbital rhabdomyosarcoma | R. deltoid | Male | 5 |
| [41] | Testicular seminoma | R. femoral | Male | 62 |
| [42] | Urothelial bladder cancer | L. obturator internus | Male | 65 |
| [43] | Neuroendocrine tumor | L. vastus intermedius | Male | 66 |
| [44] | Gestational Choriocarcinoma | R. medial rectus | Female | 30 |
| [45] | Oesophageal cancer | R. infraspinatus, L. psoas, L. gluteus medius, L. iliopsoas | Male | 53 |
| [46] | Urothelial cell carcinoma | L. sartorius | Male | 45 |
| [47] | Oropharyngeal Squamous Cell Carcinoma | R. scalene, L. hamstring | Male | 48 |
| [48] | Squamous cell lung cancer | L. adductor longus | Male | 64 |
| [49] | Breast carcinoma | L. vastus medialis | Female | 37 |
| [50] | Hepatocellular carcinoma | L. deltoid, L. trapezius, L. infraspinatus, R. subscapular, R. Latissimus dorsal | Female | 68 |
| [51] | Rectosigmoid adenocarcinoma | L. psoas | Female | 52 |
| [52] | Oesophageal Cancer | extensor digitorum communis, thumb adductor, ulnar extensors | Female | 77 |
| [53] | Gastric adenocarcinoma | Rectus abdominis | Male | 75 |
| [54] | Renal Cell Carcinoma | Splenius cervicis | Male | 70 |
| [55] | Hepatocellular carcinoma | Intercostal | Male | 65 |
| [56] | Gallbladder carcinoma | R. masseter, R. quadriceps femoris | Male | 55 |
| [57] | Gallbladder cancer | L. medium gluteus, para-spinal | Male | 76 |
| [58] | Non small-cell lung cancer | R. medial thigh | Male | 45 |
| [59] | Pancreatic cancer | R./ L. psoas, R./L. iliopsoas | Male | 67 |
| [60] | Squamous Cell Carcinoma of the Bladder | R. gluteus maximus, R. erector spinae, cardiac | Female | 55 |
| [61] | Gastric cancer | L. medial rectus | Female | 54 |
| [62] | Melanoma of unknown primary | R. temporalis | Female | 75 |
| [63] | Urothelial cell carcinoma | R. flexor digitorum superficialis, flexor digitorum profundus | Male | 76 |
| [64] | Unknown primary | L. psoas | Female | 70 |
| [65] | Breast cancer | Sternocleidomastoid | Female | 84 |
| [66] | Lung squamous cell cancer | R. Triceps | Male | 62 |
| [67] | Pancreatic adenocarcinoma | L. trapezius, R./L. deltoids, L. pectoralis major, L. triceps, paraspinal, R./L. piriformis, R. iliopsoas | Female | 62 |
| [68] | Pulmonary Adenocarcinoma | R. inferior trapezius, paraspinous | Male | 39 |
| [69] | Lung squamous cell carcinoma | L. gastrocnemius | Male | 70 |
| [70] | Bladder carcinoma | R. obturator, R. adductor, R. quadriceps femoris | Male | 60 |
| [71] | Cervical adenocarcinoma | Psoas | Female | 59 |
| [72] | Colon adenocarcinoma | R. brachialis | Male | 66 |
| [73] | Squamous Cell Carcinoma of the Lung | R. temporalis, cardiac | Female | 72 |
| [74] | Urothelial carcinoma of the bladder | R./ L. psoas major, quadratus lumborum, iliacus, gluteus medius/minimus, obturator externus, quadratus femoris, adductor brevis/magnus | Male | 83 |
| [75] | Hepatocellular carcinoma | R. superior oblique, lateral rectus, inferior rectus, L. lateral and medial rectus | Male | 44 |
| [76] | Urothelial cell carcinoma | R. deltoid | Male | 65 |
| [77] | Gastro-oesophageal adenocarcinoma | R. inferior rectus, R./L. medial recti, L. superior rectus, R. medial rectus | Male | 77 |
| [78] | Urothelial carcinoma | L. iliopsoas, piriformis, gluteus maximus, obturator internus | Male | 45 |
| [79] | Pulmonary squamous cell carcinoma | R. thenar | Male | 76 |
| [80] | Uterine myoma | L. rectus abdominis | Female | 48 |
| [81] | Neuroendocrine tumor | L./ R. extraocular | Female | 45 |
| [82] | Gastric adenocarcinoma | L. latissimus dorsi | Male | 47 |
| [83] | Renal cell carcinoma | R. masseter | Male | 71 |
| [84] | Cervical carcinoma | L. biceps | Female | 50 |
| [85] | Transitional cell carcinoma of bladder | R. adductor brevis, quadriceps femoris, gastrocnemius, L. quadriceps, semitendinosus | Male | 64 |
| [86] | Muscle Invasive Bladder Cancer | L. sartorius | Male | 51 |
| [87] | Malignant pheochromocytoma | R. inferior rectus | Male | 47 |
| [88] | Breast cancer | Sternocleidomastoid | Female | 84 |
| [89] | Invasive ductal carcinoma | Rectus abdominis | Female | 51 |
| [89] | Invasive ductal carcinoma | R. gluteus maximus | Female | 52 |
| [90] | Renal carcinoid tumor | L. medial rectus | Male | 57 |
| [91] | Tongue carcinoma | L. vastus lateralis | Male | 50 |
| [92] | Breast carcinoma | R. medial rectus | Female | 63 |
| [93] | Transitional cell carcinoma of the urinary bladder | R. adductor longus | Male | 62 |
| [94] | Gastric adenocarcinoma | Latissimus dorsi, transverse abdominis, iliacus, iliopsoas, rectus femoris | Male | 71 |
| [95] | Prostate cancer | L. psoas | Male | 61 |
| [96] | Pleural mesothelioma | Rhomboid | Female | 56 |
| [97] | Gastric cancer | R. gluteal | Male | 64 |
| [98] | Hepatocellular carcinoma | L. Soleus | Male | 76 |
| [99] | Lung cancer | Trapezius | Male | 53 |
| [99] | Squamous cell carcinoma | Adductor | Male | 59 |
| [99] | Lung cancer | R. iliac | Male | 56 |
| [100] | Bronchopulmonary cancer | L. psoas | Male | 40 |
| [101] | Lung carcinoma | R. vastus intermedius | Female | 45 |
| [102] | Colon carcinoma | Deltoid, sternocleidomastoid, trapezius, infraspinatus, adductor magnus | Female | 71 |
| [103] | Renal cell carcinoma | Temporalis | Male | 51 |
| [104] | Intrahepatic cholangiocarcinoma | R. psoas, R. paraspinous | Female | 55 |
| [105] | Breast cancer | Gluteus maximus | Female | 53 |
| [106] | Oesophageal cancer | R. para-vertebral | Male | 65 |
| [107] | Cervical cancer | Abdominal wall | Female | 51 |
| [108] | Hilar cholangiocarcinoma | R. psoas, iliacus | Female | 69 |
| [109] | Peritoneal mesothelioma | Lateral abdominal, adductor group, R. quadratus lumborum | Male | 49 |
| [110] | Papillary thyroid carcinoma | R./L. piriformis, L. erector spinae, gluteus maximus | Male | 84 |
| [111] | Metaplastic breast carcinoma | L. proximal forearm, R. psoas/ quadratus lumborum | Male | 65 |
| [112] | Renal cell carcinoma | Biceps femoris | Male | 58 |
| [113] | Non-small cell lung carcinoma | L. median gluteus | Female | 45 |
| [114] | Lung adenocarcinoma | Erector spinae | Male | 71 |
| [115] | Esophageal adenocarcinoma | R. psoas | Male | 49 |
| [116] | Urothelial carcinoma | R. vastus lateralis | Male | 71 |
| [117] | Lung adenocarcinoma | R. gastrocnemius | Female | 77 |
| [118] | Neuroendocrine tumor | Medial rectus | Female | 72 |
| [119] | Parotid pleomorphic adenoma | R. supraspinatus | Female | 65 |
| [120] | Cervical cancer | R. latissimus dorsi | Female | 57 |
| [121] | Mon-small cell lung cancer | L. forearm extensors | Male | 39 |
| [122] | Melanoma | L. triceps | Male | 50s |
| [123] | Hepatocellular carcinoma | R. semimembranosus | Female | 35 |
| [124] | Gastrointestinal malignant tumor | R. subscapularis | Female | 69 |
| [125] | Breast carcinoma | R. superior, lateral and inferior recti, and the L. superior rectus | Female | 61 |
| [126] | Gastrointestinal stromal tumor | R. tibialis anterior | Female | 66 |
| [127] | Hepatocellular Carcinoma | R. teres major | Male | 78 |
| [128] | Adrenal cortical carcinoma | L. infraspinatus, L. serratus anterior, L. subscapularis | Male | 47 |
| [129] | Gastric diffuse adenocarcinoma | R./L. gluteal, posterior thigh | Not-specified | 62 |
| [130] | Breast cancer | L. oblique (eye) | Female | 46 |
| [131] | Endometrioid adenocarcinoma | Deltoid | Female | 69 |
| [132] | Breast carcinoma | R. external oblique, internal oblique, transverse abdominal, and iliac | Female | 49 |
| [133] | Non-small cell lung carcinoma | Orbital, pectoralis | Male | 63 |
| [134] | Cervical cancer | R. oblique | Female | 75 |
| [135] | Cervical cancer | R. gluteal | Female | 56 |
| [136] | Hepatocellular carcinoma | R. biceps femoris | Male | 81 |
| [137] | Choriocarcinoma | Lumbar | Female | 34 |
| [138] | Breast cancer | L. external rectus | Female | 58 |
| [139] | Intrahepatic cholangiocarcinoma | R. thoracic , L. buttock | Male | 45 |
| [140] | Non-small Cell Lung cancer | L. gluteus | Male | 57 |
| [141] | Gastric carcinoma | R. adductor, L. gluteal | Male | 67 |
| [142] | Anaplastic thyroid cancer | R. gluteal s, R./L. iliopsoas, deltoid | Male | 44 |
| [143] | Renal cell carcinoma | R. psoas , R. paravertebral , R. gluteus medius | Male | 48 |
| [144] | Non-small Cell Lung Carcinoma | L. gluteus, L., R. supraspinatus, L. psoas | Female | 45 |
| [145] | Renal Cell Carcinoma | R. masseter | Male | 76 |
| [146] | Hepatocellular carcinoma | L./R. erector spinae R. psoas , L. rectus abdominis | Male | 54 |
| [147] | Invasive ductal carcinoma | L. inferior rectus | Female | 63 |
| [148] | Cervical cancer | L. paraspinus | Female | 34 |
| [149] | Gastric cancer | R. gluteal | Male | 64 |
| [150] | Renal carcinoma | R. erector spinae | Male | 74 |
| [151] | Esophageal squamous cell carcinoma | R. erector spinae | Male | 70 |
| [152] | Osteosarcoma | R. trapezius | Male | 51 |
| [153] | Rectal polyp | L. piriformis | Female | 67 |
| [154] | Breast cancer | R. rectus abdominus | Female | 58 |
| [155] | Renal cell carcinoma | L. Soleus | Male | 74 |
| [156] | Cystosarcoma phyllodes | Pectoralis major | Female | 45 |
| [157] | Lung adenocarcinoma | R. Bicep | Female | 49 |
| [158] | Micropapillary carcinoma of thyroid | L. deltoid | Male | 66 |
| [159] | Gastrointestinal stromal tumors | R. adductor magnus | Male | 78 |
| [160] | Gastric Adenocarcinoma | R. Superior Oblique | Male | 53 |
| [161] | Ductal breast carcinoma | Lateral rectus and inferior rectus | Female | 43 |
| [162] | Cystosarcoma phyllodes tumor | L. Soleus | Female | 25 |
| [163] | Retroperitoneal fibrosis | L. iliopsoas | Female | 46 |
| [164] | Pancreatic neuroendocrine tumors | L. calf | Male | 34 |
| [165] | Pancreatic cancer | Trapezius , teres minor , infraspinatus | Female | 72 |
| [166] | Pancreatic adenocarcinoma | L. rectus abdominis | Female | 63 |
| [167] | Breast cancer | R. pectoral | Female | 74 |
| [168] | Squamous cell carcinoma of the cervix | R. intercostal | Female | 43 |
| [168] | Invasive squamous carcinoma of the cervix | R. forearm | Female | 39 |
| [169] | Pancreatic cancer | L. iliac | Male | 61 |
| [170] | Transitional cell bladder carcinoma | L. gluteal | Male | 66 |
| [171] | Follicular thyroid carcinoma | Posterior thigh | Female | 42 |
| [172] | Non-small cell carcinoma | L. Psoas | Male | 75 |
| [172] | Non-small cell carcinoma | R. psoas | Female | 66 |
| [172] | Non-small cell carcinoma | L. Psoas | Female | 34 |
| [173] | Melanoma | R. lateral rectus | Male | 47 |
| [174] | Gastrointestinal stromal tumor | L. quadriceps, gluteal, paravertebral | Male | 54 |
| [175] | Hepatocellular carcinoma | Paravertebral | Male | 55 |
| [176] | Thymoma | R. semimembranosus | Male | 84 |
| [177] | Colorectal cancer | Cervical skeletal | Male | 83 |
| [178] | Lung squamous cell carcinoma | L. iliac, L. gluteal , L. internal/external oblique | Male | 60 |
| [179] | Non-small cell lung cancer | R. quadratus femoris vastus lateralis | Male | 48 |
| [179] | Non-small cell lung cancer | R. gluteus maximus | Male | 50 |
| [180] | Esophageal Carcinoma | L. medial rectus | Not-specified | 73 |
| [181] | Renal cell carcinoma | R. gluteus maximus | Male | 67 |
| [181] | Renal cell carcinoma | R. gluteus medius | Male | 74 |
| [182] | Follicular thyroid carcinoma | R. gluteas medius | Female | 45 |
| [183] | Renal cell carcinoma | L. deltoid | Male | 7 |
| [184] | Testicular rhabdomyosarcoma | Anterior papillary | Male | 30 |
| [185] | Esophageal carcinoma | Temporalis | Female | 62 |
| [186] | Thyroid carcinoma | L. adductor | Male | 69 |
| [187] | Cervical cancer | L. biceps | Female | 52 |
| [188] | Lung cancer | L. levator scapulae | Female | 67 |
| [189] | Adenocarcinoma cervix | R./L. deltoid, R./L. paraspinal, L. trapezius , R./L. supraspinatus , R./L. biceps, R./L. triceps brachii, L. trapezius, R./L. infraspinatus, R./L. gluteal , R./L. pyriformis, R. obturator internus, L. rectus femoris | Female | 48 |
| [190] | Sacrococcygeal chordoma | R. gluteus Maximus, R. pectineus, R. deltoid, R. triceps brachii | Male | 50 |
| [191] | Squamous cell carcinoma of larynx | R. gluteus medius | Male | 57 |
| [192] | Gastrointestinal Carcinoid Tumor | R./L. extraocular | Male | Early 70s |
| [193] | Lung Adenocarcinoma | R. trapezius | Female | 76 |
| [194] | Papillary thyroid carcinoma | Buttock | Female | 65 |
| [195] | Papillary thyroid cancer | L. Gastrocnemius | Male | 31 |
| [196] | Pulmonary adenocarcinoma | R. vastus lateralis, L. biceps femoris , L. gluteus minimus , L. paraspinal | Male | 71 |
| [197] | Anaplastic thyroid cancer | L. gluteal | Female | 61 |
| [198] | Pancreatic ductal adenocarcinoma | L. external abdominal oblique | Male | 46 |
| [199] | Papillary thyroid cancer | L. rectus abdominis | Male | 53 |
| [200] | Presacral carcinoid tumor | Gluteal | Female | 48 |
| [201] | Gastrointestinal stromal tumor | L. medial thigh | Male | 59 |

**References for case studies**

1. Abdinejad, M., & Alavi, M. (2023). 18F‐FDG PET/CT imaging of several asymptomatic skeletal muscle metastases of mixed germ cell ovarian cancer. *Clinical Case Reports, 11*(6), doi:10.1002/ccr3.7251.

2. Agrawal, K., Bhattacharya, A., Singh, N., Harisankar, C. N. B., & Mittal, B. R. (2013). Skeletal muscle metastases as the initial manifestation of an unknown primary lung cancer detected on F-18 fluorodeoxyglucose positron emission tomography/computed tomography. *Indian journal of nuclear medicine, 28*(1), 34, doi:10.4103/0972-3919.116814.

3. Aguirre, L., Salcedo, J., Zuquello, R., Garcia-Buitrago, M., & Ardalan, B. (2019). Metastatic involvement of skeletal muscle from gastric adenocarcinoma. *Oxford medical case reports, 2019*(8), omz081, doi:10.1093/omcr/omz081.

4. Akutsu, N., Kawakami, Y., Numata, Y., Hirano, T., Wagatsuma, K., Ishigami, K., et al. (2022). A case of hepatocellular carcinoma with long-term survival by multidisciplinary treatment for cranial and skeletal muscle metastases. *Clinical Journal of Gastroenterology, 15*(5), 960-967, doi:10.1007/s12328-022-01669-7.

5. Albulescu, D.-M., Ionovici, N., Moldovan, H.-R., Demetrian, A.-D., Bălă, V.-S., Constantin, C., et al. (2017). Muscle metastases from cervical carcinoma-case report. *Rom J Morphol Embryol, 58*(2), 545-551.

6. Almusarhed, M., & Eldeeb, H. (2017). Solitary biceps muscle metastasis from breast cancer. *BMJ case reports, 2017*, bcr-2017-220597, doi:10.1136/bcr-2017-220597.

7. Amir, G. J., & Juweid, M. E. (2010). Nasopharyngeal squamous cell carcinoma metastatic to psoas muscle. *Clinical nuclear medicine, 35*(7), 545-546, doi:10.1097/RLU.0b013e3181e05daa.

8. Andrianandrasana, N. O. T. F., Randrianarisoa, R. M. F., Navoly, P., Ranaivoson, M. A. C., Vololontiana, H. M. D., & Rafaramino, F. (2023). Melanoma of unknown primary origin with skeletal muscle metastasis: a case report. *Journal of Medical Case Reports, 17*(1), 90, doi:10.1186/s13256-023-03813-4.

9. Asha, S., Yasmin, E., & Stastny, J. F. (2019). Lobular breast carcinoma metastasis to skeletal muscle, two case reports diagnosed by ultrasound guided FNA with evaluation of the roles of interventional cytopathology. *Diagnostic cytopathology, 47*(3), 222-225, doi:10.1002/dc.24046.

10. Atik, M., Abarbanel, D. N., & Sener, U. (2022). Teaching NeuroImage: Horizontal Diplopia Due to Extraocular Muscle Metastasis. *Neurology, 99*(15), 669-670, doi:10.1212/Wnl.0000000000201061.

11. Azadeh, P., Joybari, A. Y., Sarbaz, S., Ghiasi, H. A., & Farasatinasab, M. (2016). Solitary psoas muscle metastasis of gastroesphageal junction adenocarcinoma. *Iranian Journal of Pathology, 11*(1), 76.

12. Bae, S. Y., Lee, S. K., Koo, M. Y., Hur, S. M., Choi, M.-Y., Cho, D. H., et al. (2011). Distant, solitary skeletal muscle metastasis in recurrent papillary thyroid carcinoma. *Thyroid, 21*(9), 1027-1031, doi:10.1089/thy.2010.0249.

13. Banerjee, D., Raghavapuram, S., George, N. E., Korourian, S., Fedda, F. A., Perisetti, A., et al. (2018). Cholangiocarcinoma Presenting as Linitis Plastica with Unusual Metastases to the Psoas Muscle and Urinary Bladder. *ACG case reports journal, 5*, doi:10.14309/crj.2018.51.

14. Banzo, I., Martínez-Rodriguez, I., Quirce, R., Jimenez-Bonilla, J., & Carril, J. M. (2011). Asymptomatic muscle metastasis from pulmonary adenocarcinoma. *Thorax, 66*(7), 642-642, doi:10.1136/thx.2010.155903.

15. Bashir, U., Qureshi, A., Khan, H. A., & Uddin, N. (2011). Gastrointestinal stromal tumor with skeletal muscle, adrenal and cardiac metastases: an unusual occurrence. *Indian Journal of Pathology Microbiology, 54*(2), 362, doi:10.4103/0377-4929.81638.

16. Basu, S., & Mahajan, A. (2014). Psoas muscle metastasis from cervical carcinoma: Correlation and comparison of diagnostic features on FDG-PET/CT and diffusion-weighted MRI. *World journal of radiology, 6*(4), 125, doi:10.4329/wjr.v6.i4.125.

17. Belbarka, R., Fadoukheir, Z., Delafouchardiere, C., Desseigne, F., & Errihani, H. (2014). An unusual presentation of pancreatic cancer: muscular metastasis. *Indian journal of cancer, 51*(3), 369-370, doi:10.4103/0019-509X.146714.

18. Bello-Roufai, D., Soares, D. G., Kerrou, K., Khalil, A., Richard, S., Gligorov, J., et al. (2017). Long-term complete response in a breast cancer patient with skeletal muscle metastases diagnosed using 18F-FDG-PET. *Oxford medical case reports, 2017*(2), omx002, doi:10.1093/omcr/omx002.

19. Benzalim, M., Ouassil, S., Alj, S., Boutakioute, B., Hadri, K., Idrissi, M. O., et al. (2023). Ossifying skeletal muscle metastasis as an initial manifestation of colorectal adenocarcinoma. *Radiology Case Reports, 18*(6), 2264-2267, doi:10.1016/j.radcr.2023.02.041.

20. Berdasco, K. F., Díaz, L. F., Jiménez-Fonseca, P., Blanco, J. C., & Montanés, C. B. (2019). Unilateral exophthalmos secondary to esophageal adenocarcinoma metastasis to the medial rectus muscle. *Archivos de la Sociedad Española de Oftalmología, 94*(10), 510-513, doi:10.1016/j.oftal.2019.07.003.

21. Bhoil, A., Mittal, B. R., Bhattacharya, A., Rane, S., Nijhawan, R., & Gupta, V. (2013). Renal cell carcinoma presenting as isolated deltoid muscle metastasis 12 years after radical nephrectomy detected on 18F-FDG PET/CT. *Clinical nuclear medicine, 38*(12), e474-476, doi:10.1097/RLU.0b013e31827a24e9.

22. Camnasio, F., Scotti, C., Borri, A., Fontana, F., & Fraschini, G. (2010). Solitary psoas muscle metastasis from renal cell carcinoma. *ANZ journal of surgery, 80*(6), 466-467, doi:10.1111/j.1445-2197.2009.05195.x.

23. Caobelli, F., Paghera, B., Panarotto, M. B., Camoni, L., & Giubbini, R. (2011). Two distant muscular metastases from papillary carcinoma of the thyroid demonstrated by 18 F-FDG PET/CT and confirmed by biopsy. *Nuclear medicine molecular imaging, 45*(4), 324-325, doi:10.1007/s13139-011-0112-x.

24. Capek, S., Amrami, K. K., Howe, B. M., & Spinner, R. J. (2015). Perineural tumor spread to the muscle: An alternative for muscle metastasis? *Clinical Anatomy, 28*(5), 560-562, doi:10.1002/ca.22485.

25. Cappellani, A., Di Vita, M., Menzo, E. L., Zanghì, A., Lanzafame, S., Veroux, P., et al. (2011). Muscular metastasis from mesocolic and duodenal leiomyosarcoma. *Ann. Ital. Chir, 82*(5), 383-387.

26. Carey, K., Bestic, J., Attia, S., Cortese, C., & Jain, M. (2014). Diffuse skeletal muscle metastases from sacral chordoma. *Skeletal Radiology, 43*(7), 985-989, doi:10.1007/s00256-013-1794-1.

27. Casas, M. G. E., Rosario, M., Battad, G., Mercado, A. C., Hermogenes, T. A., Hernandez, A., et al. (2021). Challenge of treating skeletal muscle metastasis during the COVID-19 pandemic in a low-resource setting. *ecancermedicalscience, 15*, 1235, doi:10.3332/ecancer.2021.1235.

28. Celotto, F., Tropea, S., Rastrelli, M., Saibene, T., Gazzetta, G., Dieci, M. V., et al. (2021). Solitary biceps muscle metastasis from neuroendocrine breast tumor. *The Breast Journal, 27*(2), 185-187, doi:10.1111/tbj.14139.

29. Ceriani, L., Treglia, G., Paone, G., Bongiovanni, M., Franscella, S., Giovanella, L. J. T. J. o. C. E., et al. (2013). Unusual muscular metastases from papillary thyroid carcinoma detected by fluorine-18-fluorodeoxyglucose PET/MRI. *The Journal of Clinical Endocrinology Metabolism, 98*(6), 2208-2209, doi:10.1210/jc.2013-1472.

30. Cherraqi, A., Mandour, J. E., Messaoud, O., Benameur, Y., Tanz, R., El Fenni, J., et al. (2022). Unusual finding of bronchopulmonary carcinoma through a pterygoid muscle metastasis. About a case. *Radiology Case Reports, 17*(9), 3373-3376, doi:10.1016/j.radcr.2022.06.039.

31. Chisthi, M. M., & Manju, P. (2013). Skeletal muscle metastases from pancreatic carcinoma–A case report and review of literature. *Indian Journal of Surgery, 75*(3), 181-184, doi:10.1007/s12262-012-0650-1.

32. Choi, S. A., Choi, E. J., & Park, H. S. (2018). Interesting dynamic contrast-enhanced magnetic resonance imaging findings of metaplastic breast carcinoma with skeletal muscle metastasis. *Chinese Medical Journal, 131*(07), 881-882, doi:10.4103/0366-6999.228228.

33. Cichowitz, A., Thomson, B., & Choong, P. (2011). GIST metastasis to adductor longus muscle. *ANZ journal of surgery, 81*(6), 490-491, doi:10.1111/j.1445-2197.2011.05766.x.

34. Ciurea, A. I., Boca, I., Rogojan, L., Ciule, L. D., & Ciortea, C. A. (2019). Pectoralis muscle metastases from breast cancer in a young patient detected by automated breast ultrasound. *Medical ultrasonography, 21*(2), 200-203, doi:10.11152/mu-1769.

35. Crombé, A., Lintingre, P.-F., Le Loarer, F., Lachatre, D., & Dallaudière, B. (2018). Multiple skeletal muscle metastases revealing a cardiac intimal sarcoma. *Skeletal Radiology, 47*(1), 125-130, doi:10.1007/s00256-017-2768-5.

36. D'Elia, C., Cai, T., Luciani, L., Bonzanini, M., & Malossini, G. (2013). Pelvic and muscular metastasis of a renal cell carcinoma: A case report. *Oncology letters, 5*(4), 1258-1260, doi:10.3892/ol.2013.1172.

37. Dalle Carbonare, M., Goh, M. X., AlshiekhAli, Z., & Howlett, D. (2017). Metastatic melanoma of unknown primary in the temporalis muscle. *BMJ case reports, 2017*, bcr-2017-221577, doi:10.1136/bcr-2017-221577.

38. Dandroo, J. M., Mohsin, N., & Rather, S. A. (2017). Isolated skeletal muscle metastasis and hypercalcemia in non-small cell lung carcinoma. *Saudi journal of medicine medical sciences, 5*(1), 62, doi:10.4103/1658-631X.194245.

39. Daneti, D., Senthamizhselvan, K., Verma, S. K., & Mohan, P. (2021). Gastric adenocarcinoma presenting with multiple skeletal muscle metastases. *BMJ Case Reports CP, 14*(1), e239518, doi:10.1136/bcr-2020-239518.

40. Das, J. K., Tiwary, B., Paul, S., Bhattacharjee, H., Cida, B., & Das, D. (2010). Primary orbital rhabdomyosarcoma with skeletal muscle metastasis. *Oman Journal of Ophthalmology, 3*(2), 91-93, doi:10.4103/0974-620X.64235.

41. Degirmencioglu, S., & Degirmencioglu, B. (2013). Isolated femoral muscle and bone metastases rarely encountered in testicular seminoma. *Case Reports in Oncological Medicine, 2013*, 780493, doi:10.1155/2013/780493.

42. Dell'Atti, L. (2015). A rare metastatic myositis ossificans of obturator muscle secondary to urothelial carcinoma. *Rare Tumors, 7*(3), 108-110, doi:10.4081/rt.2015.5870.

43. delos Santos-Cabalona, W., Kozyreva, O. N., Davidoff, A., Wolfe, G., & Hackford, A. (2013). Skeletal Muscle Metastases in a Patient With Neuroendocrine Tumor. *World journal of oncology, 4*(2), 114, doi:10.4021/wjon609w.

44. Dhrami-Gavazi, E., Lo, C., Patel, P., Galic, V., Pareja, F., & Kazim, M. (2014). Gestational choriocarcinoma metastasis to the extraocular muscle: a case report. *Ophthalmic Plastic Reconstructive Surgery, 30*(3), e75-e77, doi:10.1097/IOP.0b013e31829bb2a7.

45. Domínguez, M., Rayo, J., Serrano, J., Infante, J., Garcia, L., & Moreno, M. (2016). Uncommon isolated distant subcutaneous tissue and skeletal muscle metastasis from oesophageal cancer diagnosed by 18F-FDG PET/CT. *Revista Española de Medicina Nuclear e Imagen Molecular, 35*(1), 38-41, doi:10.1016/j.remn.2015.07.005.

46. Doo, S. W., Kim, W. B., Kim, B. K., Yang, W. J., Yoon, J. H., Song, Y. S., et al. (2012). Skeletal muscle metastases from urothelial cell carcinoma. *Korean journal of urology, 53*(1), 63-66, doi:10.4111/kju.2012.53.1.63.

47. Drew, Z. J. (2018). Oropharyngeal Squamous Cell Carcinoma Metastasis to Distal Skeletal Muscle on FDG PET/CT. *Clinical nuclear medicine, 43*(11), e402-e403, doi:10.1097/RLU.0000000000002262.

48. Du, L., & Sun, Y. (2019). Skeletal muscle metastasis from squamous cell lung cancer was first found by ultrasound: a case report. *Translational Cancer Research, 8*(8), 2936, doi:10.21037/tcr.2019.12.55.

49. Erdemir, R. U., & Elmas, Ö. (2021). PET/CT Findings of a Patient with Striped Muscle Metastasis of Invasive Breast Carcinoma. *Molecular Imaging Radionuclide Therapy, 30*(2), 117, doi:10.4274/mirt.galenos.2020.90377.

50. Evangelista, L., Giacomuzzi, F., Di Gregorio, F., & Rensi, M. (2021). Skeletal Muscle Metastases in HCC Revealed by 18F-Choline PET/CT. *Clinical nuclear medicine, 46*(12), e592-e593, doi:10.1097/RLU.0000000000003715.

51. Farraj, K., Im, J., Gonzalez, L. F., Lu, A., Portnoy III, R., & Podrumar, A. (2021). Solitary cystic psoas muscle metastasis from rectosigmoid adenocarcinoma. *Journal of Investigative Medicine High Impact Case Reports, 9*, 23247096211024067, doi:10.1177/23247096211024067.

52. Fujimoto, Y., Nakashima, Y., Sasaki, S., Jogo, T., Hirose, K., Edahiro, K., et al. (2017). Chemoradiotherapy for solitary skeletal muscle metastasis from oesophageal cancer: case report and brief literature review. *Anticancer research, 37*(10), 5687-5691, doi:10.21873/anticanres.12005.

53. Fukui, Y., Kubo, N., Sakurai, K., Tamamori, Y., Maeda, K., & Ohira, M. (2021). Metachronous port site, muscular and subcutaneous metastases from a gastric adenocarcinoma: a case report and review of articles. *Surgical Case Reports, 7*(1), 124, doi:10.1186/s40792-021-01202-x.

54. Fuoco, V., Barisella, M., Lorenzoni, A., Verzoni, E., & Maccauro, M. (2022). Unexpected Detection of Skeletal Muscle Renal Cell Carcinoma Metastasis With 99mTc-EDDA/HYNIC-Tyr3-Octreotide (Tektrotyd) Scan. *Clinical nuclear medicine, 47*(12), e762-e764, doi:10.1097/Rlu.0000000000004302.

55. Furumoto, K., Miura, K., Nagashima, D., Kojima, H., Mori, T., Ito, D., et al. (2012). Solitary metastasis to the intercostal muscle from hepatocellular carcinoma: a case report. *International Journal of Surgery Case Reports, 3*(7), 322-326, doi:10.1016/j.ijscr.2012.04.003.

56. Ghosh, J., Ganguly, S., Dabkara, D., Biswas, B., Chatterjee, A., Mukhopadhyay, S., et al. (2019). Metachronous muscle metastasis in a case of metastatic gallbladder cancer with TP35 gene mutation: a rare case report. *South Asian Journal of Cancer, 8*(04), 240-257, doi:10.4103/sajc.sajc_139_19.

57. Gilardi, L., & Paganelli, G. (2011). Muscle metastases from gallbladder cancer. *ecancermedicalscience, 5*, 236, doi:10.3332/ecancer.2011.236.

58. Giugliano, F. M., Alberti, D., Guida, G., Palma, G. D., Iadanza, L., Mormile, M., et al. (2013). Non small-cell lung cancer with metastasis to thigh muscle and mandible: two case reports. *Journal of Medical Case Reports, 7*(1), 1-5, doi:10.1186/1752-1947-7-98.

59. Gong, W., Yang, X., Wu, J., Ou, L., & Zhang, C. (2022). 68Ga-FAPI PET/CT imaging of multiple muscle metastases of pancreatic cancer. *Clinical nuclear medicine, 47*(1), 73-75, doi:10.1097/RLU.0000000000003888.

60. Gorospe, L., García-Santana, E., Jane-Soler, P., del Val Gómez-Martínez, M., Gómez-Dos-Santos, V., García-Gómez-Muriel, I., et al. (2017). Cardiac and Skeletal Muscle Metastases From Squamous Cell Carcinoma of the Bladder: Positron Emission Tomography-Computed Tomography Findings. *Urology, 100*, e5-e6, doi:10.1016/j.urology.2016.11.030.

61. Goto, S., Takeda, H., Sasahara, Y., Takanashi, I., & Yamashita, H. (2019). Metastasis of advanced gastric cancer to the extraocular muscle: a case report. *Journal of Medical Case Reports, 13*(1), 1-3, doi:10.1186/s13256-019-2031-x.

62. Grech, A., Mercieca, N., Calleja-Agius, J., & Abela, R. (2020). Metastatic malignant melanoma of unknown primary in temporalis muscle. *Journal of Surgical Case Reports, 2020*(6), rjaa202, doi:10.1093/jscr/rjaa202.

63. Guidi, M., Fusetti, C., & Lucchina, S. (2016). Skeletal muscle metastases to the flexor digitorum superficialis and profundus from urothelial cell carcinoma and review of the literature. *Case reports in urology, 2016*, 2387501, doi:10.1155/2016/2387501.

64. Gunn, C., & Fani, M. (2022). Psoas muscle metastatic disease mimicking a psoas abscess on imaging. *BMJ Case Reports CP, 15*(8), e250654, doi:10.1136/bcr-2022-250654.

65. Gyorffy, J., Philbrick, S. M., Bersabe, A. R., Upton, R. J., Mathis, D. A., Peters, A., et al. (2017). A unique case of muscle-invasive metastatic breast cancer mimicking myositis. *Case Reports in Oncological Medicine, 2017*, 2648296, doi:10.1155/2017/2648296.

66. Haji, K., Sato, S., Yoneda, H., Nisisho, T., Nokihara, H., & Nishioka, Y. (2021). Multidisciplinary treatment of skeletal muscle metastasis from lung cancer: A case of triceps muscle metastasis of lung squamous cell cancer. *Respiratory Medicine Case Reports, 33*, 101470, doi:10.1016/j.rmcr.2021.101470.

67. Harmon, C. E., Lamsal, S. P., Harmon, T. S., Mohamed, K., Meyer, T. E., Harmon, C., et al. (2021). The Path of Most Resistance: A Rare Instance of Metastatic Pancreatic Adenocarcinoma Identified Within Skeletal Muscle. *Cureus, 13*(3), doi:10.7759/cureus.13947.

68. Harrison, M., Jones, A., & Abebe, A. (2018). Pulmonary Adenocarcinoma Presenting as Paraspinal Muscle Metastatic Mass. *Case Reports in Oncological Medicine, 2018*, doi:10.1155/2018/5719382.

69. Hirst, B., & Powell, J. (2018). Muscle metastases: rare problem with unusual solution. *BMJ supportive palliative care, 8*(3), 297-298, doi:10.1136/bmjspcare-2018-001491.

70. Honda, Y., Sekine, T., Kimata, R., Motoda, N., Takahashi, K., Yamane, A., et al. (2023). Early and post-treatment imaging findings in perineural spread: a pathway to diffuse muscle metastasis in recurrent bladder carcinoma. *Journal of Nippon Medical School*, JNMS. 2024_2091-2301, doi:10.1272/jnms.JNMS.2024_91-301.

71. Hong, J.-H., Song, S., Lee, J. K., Lee, N. W., & Lee, K. (2011). Metastatic cervical adenocarcinoma mimicking retroperitoneal sarcoma of the psoas muscle on imaging. *European journal of gynaecological oncology, 32*(2), 221.

72. Iyengar, K. P., Vinjamuri, A., & Ahuja, N. (2022). Brachialis muscle metastasis: An unusual, presenting site of metastatic adenocarcinoma of the colon. *Journal of Clinical Orthopaedics Trauma, 25*, 101771, doi:10.1016/j.jcot.2022.101771.

73. Jain, T. K., Rayamajhi, S. J., Basher, R. K., Gupta, D., Maturu, V. N., & Mittal, B. R. (2016). Disseminated skeletal muscle and cardiac metastasis from squamous cell carcinoma of the lung detected with FDG and FLT PET/CT. *World journal of nuclear medicine, 15*(3), 215, doi:10.4103/1450-1147.167607.

74. Jhang, Y.-Y., Shen, S.-H., & Wang, J.-H. (2010). Unusual presentation of urothelial carcinoma of the bladder with noncontiguous rectal and diffuse muscular skeletal metastases. *The Journal of urology, 184*(3), 1163-1164, doi:10.1016/j.juro.2010.05.067.

75. Jiang, H., Wang, Z., Xian, J., & Ai, L. (2012). Bilateral multiple extraocular muscle metastasis from hepatocellular carcinoma. *Acta radiologica short reports, 1*(1), 1-3, doi:10.1258/arsr.2011.110002.

76. Journeay, W. S., Omar, A., & Carette, S. (2013). Skeletal Muscle Metastasis Mimicking a Shoulder Effusion. *The Journal of rheumatology, 40*(9), 1616-1616, doi:10.3899/jrheum.130069.

77. Julve, M., Maviki, M., & Gillmore, R. (2014). Bilateral extraocular muscle (EOM) metastases from adenocarcinoma of the gastro-oesophageal junction (GOJ). *Case Reports, 2014*, bcr2014205368, doi:10.1136/bcr-2014-205368.

78. Kafadar, C., Kara, K., Sildiroglu, O., & Incedayi, M. (2016). Giant muscular metastasis of urothelial carcinoma presenting as a cause of extraspinal sciatica. *The Spine Journal, 16*(6), e375-e376, doi:10.1016/j.spinee.2015.12.003.

79. Kaira, K., Ayabe, E., Takahashi, T., Murakami, H., Tsuya, A., Nakamura, Y., et al. (2011). Thenar muscle metastasis as recurrence of pulmonary squamous cell carcinoma. *Asia‐Pacific Journal of Clinical Oncology, 7*(1), 15-16, doi:10.1111/j.1743-7563.2010.01373.x.

80. Kalayci, M., Gonenc, M., Yalcin, O., Kurtulus, I., Turan, I., & Avul, R. (2013). Isolated muscle metastasis of cervical cancer. *Journal of Obstetrics Gynaecology, 33*(6), 641-642, doi:10.3109/01443615.2013.797387.

81. Kamaleshwaran, K. K., Joseph, J., Upadhya, I., & Shinto, A. S. (2017). Image findings of a rare case of neuroendocrine tumor metastatic to orbital extraocular muscle in Gallium-68 DOTANOC positron emission tomography/computed tomography and therapy with Lutetium-177 DOTATATE. *Indian journal of nuclear medicine, 32*(2), 125, doi:10.4103/0972-3919.202236.

82. Kamitani, N., Watanabe, A., Kirihataya, Y., & Ko, S. (2018). Metachronous skeletal muscle metastasis without any other organ metastases after curative gastrectomy: a case report. *Surgical Case Reports, 4*, 1-5, doi:10.1186/s40792-018-0507-3.

83. Kang, S., Song, B.-I., Lee, H. J., Jeong, S. Y., Seo, J.-H., Lee, S.-W., et al. (2010). Isolated facial muscle metastasis from renal cell carcinoma on F-18 FDG PET/CT. *Clinical nuclear medicine, 35*(4), 263-264, doi:10.1097/RLU.0b013e3181d18f37.

84. Karunanithi, G., Sethi, P., Reddy, K. S., & Rani, P. R. (2010). Skeletal muscle metastasis from carcinoma cervix: a case report. *Journal of Gynecologic Oncology, 21*(3), 196-198, doi:10.3802/jgo.2010.21.3.196.

85. Kashyap, R., Mittal, B. R., Chakraborty, D., Bhattacharya, A., & Singh, B. (2010). Multiple skeletal muscle metastases in a case of transitional cell carcinoma of bladder detected by F-18 FDG PET/CT. *Nuclear medicine molecular imaging, 44*(4), 297-299, doi:10.1007/s13139-010-0052-x.

86. Katafigiotis, I., Athanasiou, A., Levis, P. K., Fragkiadis, E., Sfoungaristos, S., Ploumidis, A., et al. (2014). Metastasis to sartorius muscle from a muscle invasive bladder cancer. *Case reports in medicine, 2014*, 524757, doi:10.1155/2014/524757.

87. Khataminia, G., Talaiezadeh, A., Bagheri, A., Nazari, P., Papan, A. M., Jazayeri, N., et al. (2020). Ocular muscle metastasis as the initial presentation of a malignant pheochromocytoma: A unique case. *Clinical Case Reports, 8*(9), 1689-1692, doi:10.1002/ccr3.2990.

88. Khettab, M., Barrascout, E., & Lamuraglia, M. (2017). Sternocleidomastoid muscle metastasis of breast cancer: case report. *European journal of gynaecological oncology, 38*(1), 113-114.

89. Kim, Y. W., Seo, K. J., Lee, S. L., Kwon, K. W., Hur, J., An, H. J., et al. (2013). Skeletal muscle metastases from breast cancer: two case reports. *Journal of breast cancer, 16*(1), 117-121, doi:10.4048/jbc.2013.16.1.117.

90. Kiratli, H., Uzun, S., Tarlan, B., Ates, D., Baydar, D. E., & Söylemezoglu, F. (2015). Renal carcinoid tumor metastatic to the uvea, medial rectus muscle, and the contralateral lacrimal gland. *Ophthalmic Plastic Reconstructive Surgery, 31*(4), e91-e93, doi:10.1097/IOP.0000000000000112.

91. Kitano, H., Mamiya, A., Masuda, Y., Shinoda, K., Masaoka, Y., & Suzuki, M. (2021). A case of vastus lateralis muscle metastasis of tongue carcinoma. *JPMA. The Journal of the Pakistan Medical Association, 71*(9), 2271-2274, doi:10.47391/JPMA.01-1104.

92. Kızıloğlu, Ö. Y., Türköz, F. P., Gedar, Ö. M. T., Mestanoğlu, M., & Yapıcıer, Ö. (2019). Breast Carcinoma Metastasis to the Medial Rectus Muscle: Case Report. *Turkish journal of ophthalmology, 49*(3), 168, doi:10.4274/tjo.galenos.2018.39018.

93. Koca, I., Ucar, M., Bozdag, Z., & Alkan, S. (2014). Adductor longus muscle metastasis of transitional cell carcinoma of the urinary bladder. *Case Reports, 2014*, bcr2014203768, doi:10.1136/bcr-2014-203768.

94. Koga, Y., Baba, Y., Harada, K., Kosumi, K., Shigaki, H., Kurashige, J., et al. (2015). Multiple skeletal muscle metastases from poorly differentiated gastric adenocarcinoma. *Surgical Case Reports, 1*(1), 1-5, doi:10.1186/s40792-015-0108-3.

95. Kong, J., Sumbly, V., Idrees, Z., & Mahmood, K. (2021). An Unusual Case of Prostate Carcinoma With Metastasis to the Iliopsoas Muscle and Nerve Root Impingement. *Cureus, 13*(7), e16286, doi:10.7759/cureus.16286.

96. Kong, L., Ren, D., Chen, S., & Duan, G. (2022). A case report of breast metastasis from malignant pleural mesothelioma invading the rhabdoid muscle. *Asian Journal of Surgery, 46*(5), S1015-9584 (1022) 01572, doi:10.1016/j.asjsur.2022.11.025.

97. Korehisa, S., Kabashima, A., Ichimanda, M., Umeda, K., Koso, H., Yada, K., et al. (2021). Gluteal muscle metastasis with peritoneal dissemination from gastric cancer during postoperative adjuvant chemotherapy: a case report. *Surgical Case Reports, 7*(1), 1-6, doi:10.1186/s40792-021-01127-5.

98. Koutserimpas, C., Dargaras, N., Naoum, S., Arkoudis, N.-A., Bafaloukos, D., Kourea, H., et al. (2022). Soleus Muscle Single Metastasis from Hepatocellular Carcinoma. *Maedica, 17*(3), 714, doi:10.26574/maedica.2022.17.3.714.

99. Kwas, H. H., Zendah, I., & Ghedira, H. (2013). Skeletal muscle metastases from lung cancer. *Asian Cardiovascular Thoracic Annals, 21*(6), 741-743, doi:10.1177/0218492312470571.

100. Lahfidi, A., Zaimi, S., El Fenni, J., & Saouab, R. J. R. C. R. (2020). Low back pain indicative of psoas muscle metastasis and bronchopulmonary cancer. *Radiol Case Rep, 15*(9), 1689-1692, doi:10.1016/j.radcr.2020.06.038.

101. Lalchandani, A., Shukla, Y., Parwez, M. M., & Kumar, V. (2021). Carcinoma lung presenting with skeletal muscle metastasis: case report with review of literature. *The Surgery Journal, 7*(02), e121-e123, doi:10.1055/s-0041-1731636.

102. Landriscina, M., Gerardi, A. M. T., Fersini, A., Modoni, S., Stoppino, L. P., Macarini, L., et al. (2013). Multiple skeletal muscle metastases from colon carcinoma preceded by paraneoplastic dermatomyositis. *Case reports in medicine, 2013*, 392609, doi:10.1155/2013/392609.

103. Lee, D. W., Ryu, H. R., Kim, J. H., Choi, H. J., & Ahn, H. (2021). Isolated temporalis muscle metastasis of renal cell carcinoma. *Archives of Craniofacial Surgery, 22*(1), 66, doi:10.7181/acfs.2021.00031.

104. Lee, J., Lee, S. W., Han, S. Y., Baek, Y. H., Kim, S. Y., & Rhyou, H. I. (2015). Rapidly aggravated skeletal muscle metastases from an intrahepatic cholangiocarcinoma. *World Journal of Gastroenterology: WJG, 21*(6), 1989, doi:10.3748/wjg.v21.i6.1989.

105. Lee, J. Y., Kang, H. W., & Jung, S.-N. (2015). Solitary gluteus maximus muscle metastasis in a breast cancer patient. *Archives of Plastic Surgery, 42*(05), 661-663, doi:10.5999/aps.2015.42.5.661.

106. Leuzzi, G., Cesario, A., Margaritora, S., Parisi, A. M., Porziella, V., Meacci, E., et al. (2013). A case of oesophageal cancer with low back pain: the accidental finding of skeletal muscle metastasis. *Ann Ital Chir, 84*(2), 193-195.

107. Li, D., & Hu, X. (2022). Solitary abdominal muscle metastasis of cervical cancer after surgery mimicking mesenchymal sarcoma on MRI and 18 F-FDG PET/CT imaging. *Revista Española de Medicina Nuclear e Imagen Molecular, 41*(6), 383-384, doi:10.1016/j.remnie.2021.10.007.

108. Li, J., Henry, M. R., & Roberts, L. R. (2011). Rare distant skeletal muscle metastasis from hilar cholangiocarcinoma: report of a case. *Journal of gastrointestinal cancer, 42*(3), 171-173, doi:10.1007/s12029-010-9237-x.

109. Li, J., Yang, J., & Hu, S. (2023). Malignant Peritoneal Mesothelioma With Butterfly-Shaped Muscle Metastasis: 68Ga-FAPI PET/CT Versus 18F-FDG PET/CT. *Clinical nuclear medicine, 48*(4), 348-350, doi:10.1097/RLU.0000000000004575.

110. Li, Z.-G., Lin, Z.-C., & Mu, H.-Y. (2016). Polysplenia syndrome with splenic and skeletal muscle metastases from thyroid carcinoma evaluated by FDG PET/CT: Case report and literature review: A care-compliant article. *Medicine, 95*(4), e2532, doi:10.1097/MD.0000000000002532.

111. Liu, C. H., Chang, C., Sy, E., Lai, H.-W., & Kuo, Y.-L. (2015). Metaplastic breast carcinoma with multiple muscle metastasis: a case report. *Medicine, 94*(17), e662, doi:10.1097/MD.0000000000000662.

112. Lohiya, V., Lohiya, S., & Windsor, K. (2013). A large thigh mass: a blood clot or a rare skeletal muscle metastasis from renal cell carcinoma. *Springerplus, 2*(1), 399, doi:10.1186/2193-1801-2-399.

113. López-González, A., Huelves, M., López García, A., & Provencio, M. (2011). Skeletal muscle metastasis from NSCLC. *Journal of thoracic disease, 4*(2), 232-234, doi:10.3978/j.issn.2072-1439.2011.06.05.

114. Lu, S.-J., Rodriguez-Justo, M., Read, S., & Bomanji, J. B. (2012). Metabolic-morphologic discordant solitary skeletal muscle metastasis on [(18) F] fluorodeoxyglucose positron emission tomography/computed tomography scan in synchronous lung and esophageal cancer: a lesson. *Journal of Clinical Oncology: Official Journal of the American Society of Clinical Oncology, 30*(8), e97-e101, doi:10.1200/JCO.2011.38.9205.

115. Ludmir, E. B., Robey, B., Shelby, E., Patel-Nguyen, S. V., Rittershaus, A., & Contarino, M. R. (2016). Skeletal muscle metastasis from signet ring cell esophageal adenocarcinoma. *Translational Gastroenterology Hepatology, 1*, 37, doi:10.21037/tgh.2016.04.05.

116. Mainwaring, A., Wells, H., Banks, T., Ellul, T., & Bose, P. (2019). Skeletal Muscle Metastasis to Vastus Lateralis from a Urothelial Carcinoma: A Case Report and Review of Its Diagnosis and Management. *Case reports in urology, 2019*, 8923780, doi:10.1155/2019/8923780.

117. Matsuda, H., Hara, M., Iwakami, S.-I., & Takahashi, K. (2021). EML4-ALK positive lung adenocarcinoma with skeletal muscle metastasis in the R. calf which was treatable with lorlatinib after resistance to treatment with alectinib. *BMJ Case Reports CP, 14*(4), e240295, doi:10.1136/bcr-2020-240295.

118. Matsuo, T., Ichimura, K., Tanaka, T., Takenaka, T., & Nakayama, T. (2010). Neuroendocrine tumor (carcinoid) metastatic to orbital extraocular muscle: case report and literature review. *Strabismus, 18*(4), 123-128, doi:10.3109/09273972.2010.525779.

119. McGarry, J. G., Redmond, M., Tuffy, J. B., Wilson, L., & Looby, S. (2015). Metastatic pleomorphic adenoma to the supraspinatus muscle: a case report and review of a rare aggressive clinical entity. *Journal of Radiology Case Reports, 9*(10), 1, doi:10.3941/jrcr.v9i10.2283.

120. Miyoshi, A., Kuritani, Y., Kanao, S., Naoi, H., Otsuka, H., & Yokoi, T. (2019). Aggressive skeletal muscle metastasis from cervical cancer invading into the spinal canal: A case report. *Clinical Case Reports, 7*(2), 361, doi:10.1002/ccr3.1952.

121. Mogi, A., Kosaka, T., Yamaki, E., & Kuwano, H. (2012). Successful resection of stage IV non-small cell lung cancer with muscle metastasis as the initial manifestation: a case report. *Annals of Thoracic Cardiovascular Surgery, 18*(5), 1202280145-1202280145, doi:10.5761/atcs.cr.11.01798.

122. Mondal, A., Dingle, L., & Hough, M. (2023). Atypical late presentation of muscular metastasis of melanoma in the contralateral limb. *BMJ Case Reports CP, 16*(8), e255819, doi:10.1136/bcr-2023-255819.

123. Moon, S. Y., Han, S. Y., & Baek, Y.-H. (2022). A case report of advanced hepatocellular carcinoma treated with hepatic arterial infusion chemotherapy and sorafenib combination therapy followed by metastasectomy of lung and muscle metastases. *Journal of Liver Cancer, 22*(1), 57-62, doi:10.17998/jlc.2021.12.20.

124. Moon, Y.-L., Ahn, K. Y., Moon, S. P., Lim, S.-C., & Venkat, G. (2010). Subscapularis muscle metastases of duodenal adenocarcinoma: A case report. *Journal of shoulder elbow surgery, 19*(2), e18-e21, doi:10.1016/j.jse.2009.09.003.

125. Murthy, R., Gupta, A., Hegde, S., & Honavar, S. G. (2011). Bilateral multiple extraocular muscle metastasis from breast carcinoma. *Indian journal of ophthalmology, 59*(5), 381, doi:10.4103/0301-4738.83616.

126. Mutlu, H., Balkarlı, H., Musri, F. Y., Salim, D. K., Eryılmaz, M. K., Ünal, B., et al. (2015). Gastrointestinal stromal tumor and isolated anterior tibial muscle metastasis as first recurrence. *Journal of Cancer Research Therapeutics, 11*(4), doi:10.4103/0973-1482.151862.

127. Nakayama, A., Arai, J., Otoyama, Y., Sugiura, I., Nakajima, Y., Kajiwara, A., et al. (2022). Muscular metastasis of hepatocellular carcinoma: case report and literature review. *Internal Medicine, 61*(2), 189-196, doi:10.2169/internalmedicine.7200-21.

128. Narra, R., Syed, S., Sowjanya, N., & Veeragandham, S. (2022). Nonfunctioning adrenal cortical carcinoma with skeletal muscle metastasis: Case report and imaging at limited resource center. *Radiology Case Reports, 17*(5), 1506-1511, doi:10.1016/j.radcr.2022.02.002.

129. Nespoulous, A., Kaici, J., Michaud, J., & Nicolau, J. (2022). Multiple muscle metastases. *Joint Bone Spine, 89*(6), 105442, doi:10.1016/j.jbspin.2022.105442.

130. Nifosí, G., & Zuccarello, M. (2018). Unilateral localized extraocular muscle metastasis by lobular breast carcinoma. *BMJ case reports, 2018*, bcr-2018-224726, doi:10.1136/bcr-2018-224726.

131. Oaknin, A., Barretina, M., & Morilla, I. (2010). Muscle metastasis of low-grade endometrial carcinoma seven years after diagnosis: a case report. *Eur J Gynaecol Oncol, 31*(1), 114-116.

132. Ogiya, A., Takahashi, K., Sato, M., Kubo, Y., Nishikawa, N., Kikutani, M., et al. (2015). Metastatic breast carcinoma of the abdominal wall muscle: a case report. *Breast Cancer, 22*(2), 206-209, doi:10.1007/s12282-012-0352-3.

133. Olali, C., & Gupta, M. (2014). Simultaneous pectoralis major muscle and orbital metastasis as the primary presentation of pulmonary adenocarcinoma. *West African Journal of Medicine, 33*(1), 80-81.

134. Omokawa, N., Mabuchi, S., Iwai, K., Kawahara, N., Kawaguchi, R., Sugimoto, S., et al. (2020). Skeletal muscle metastasis as a first site of recurrence of cervical cancer: A case report and review of the literature. *Medicine, 99*(19), e20056, doi:10.1097/MD.0000000000020056.

135. Orellana, T., Ross, M., Dressen, M., Beriwal, S., & Berger, J. L. (2022). Rare presentation of metastatic cervical cancer to the R. upper extremity skeletal muscle and gluteal adipose tissue. *Radiology Case Reports, 17*(7), 2554-2558, doi:10.1016/j.radcr.2022.04.035.

136. Orita, K., Sakamoto, A., Okamoto, T., & Matsuda, S. (2019). Solitary Muscle Metastasis of Hepatocellular Carcinoma to the Biceps Femoris Muscle with Only Elevated Serum PIVKA-II: A Case Report. *The American journal of case reports, 20*, 306, doi:10.12659/AJCR.913730.

137. Pang, L., & Ma, X.-X. (2020). Choriocarcinoma with lumbar muscle metastases: A case report. *World Journal of Clinical Cases, 8*(20), 5036, doi:10.12998/wjcc.v8.i20.5036.

138. Pardines, F. H., Verdú, M. S., Vidal, A. B., Belda, J. M., & Verdú, E. M. (2019). Lateral rectus muscle biopsy as diagnosis of unknown metastatic breast cancer. *Archivos de la Sociedad Española de Oftalmología, 94*(4), 192-195, doi:10.1016/j.oftal.2018.09.002.

139. Park, S. K., Kim, Y. S., Kim, S. G., Jang, J. Y., Moon, J. H., Lee, M. S., et al. (2010). Detection of distant metastasis to skeletal muscle by 18F-FDG-PET in a case of intrahepatic cholangiocarcinoma. *The Korean journal of hepatology, 16*(3), 325, doi:10.3350/kjhep.2010.16.3.325.

140. Peravali, R., Azim, A., & Muddassir, K. (2021). Non-small Cell Lung Cancer as Skeletal Muscle Metastasis. *Journal of General Internal Medicine, 36*(11), 3573-3574, doi:10.1007/s11606-021-07099-4.

141. Pergolini, I., Crippa, S., Santinelli, A., & Marmorale, C. (2014). Skeletal muscle metastases as initial presentation of gastric carcinoma. *The American journal of case reports, 15*, 580, doi:10.12659/Ajcr.891397.

142. Piciu, D., Larg, M., Barbus, E., & Piciu, A. (2018). UNUSUAL PERITONEAL AND MUSCULAR METASTASES IN AN AGGRESSIVE AND EXTENSIVE CASE OF ANAPLASTIC THYROID CANCER ON 18F-FDG PET/CT. *Acta Endocrinologica, 14*(3), 408, doi:10.4183/aeb.2018.408.

143. Pirimoglu, B., Ogul, H., Kisaoglu, A., Karaca, L., Okur, A., & Kantarci, M. (2015). Multiple muscle metastases of the renal cell carcinoma after radical nephrectomy. *International Surgery, 100*(4), 761-764, doi:10.9738/INTSURG-D-13-00197.1.

144. Purkayastha, A., Singh, S., Bisht, N., Mishra, P. S., & Husain, A. (2018). Upfront skeletal muscle metastases from non-small cell lung carcinoma: Report of an extremely rare occurrence detected by 18F-fluorodeoxyglucose positron emission computed tomography scan. *Indian journal of nuclear medicine, 33*(4), 337, doi:10.4103/ijnm.IJNM_57_18.

145. Qin, F., Zhang, X., Zhang, J., Liu, S., Wang, Z., Xie, F., et al. (2022). Masseter Muscle Metastasis of Renal Cell Carcinoma: A Case Report and Literature Review. *Frontiers in Oncology, 12*, 830195, doi:10.3389/fonc.2022.830195.

146. Rahim, E. A., Noh, M. S., Ngah, N. A., Suraini, M. S., & Yusof, M. (2017). Hepatocellular carcinoma with disseminated skeletal muscle metastasis. *Acta radiologica open, 6*(7), 2058460117716705, doi:10.1177/2058460117716705.

147. Rich, B. J., Pasol, J., Ivan, M. E., Schaffer, M. A., Ford, J. C., & Mellon, E. A. (2022). Stereotactic Radiosurgery of Mobile Extraocular Muscle Metastasis: A Noninvasive Approach Using MRI Simulation. *Journal of neuro-ophthalmology, 42*(3), e551-e553, doi:10.1097/WNO.0000000000001477.

148. Rodriguez, J., Castro, J. C., Beltran, M., Forero, O., & Pareja, R. (2019). Simultaneous Metastasis from Cervical Cancer to the Kidney and Paraspinal Muscle: A Case Report. *Cureus, 11*(2), e4148, doi:10.7759/cureus.4148.

149. Ryu, H. R., Kim, J. H., Choi, H. J., & Ahn, H. (2021). Isolated temporalis muscle metastasis of renal cell carcinoma. *Archives of Craniofacial Surgery, 22*(1), 66, doi:10.7181/acfs.2021.00031.

150. Safadi, A., Ahmad, M. S. A., Sror, S., Schwalb, S., & Katz, R. (2018). Simultaneous metachronous renal cell carcinoma and skeletal muscle metastasis after radical nephrectomy. *Urology case reports, 16*, 17, doi:10.1016/j.eucr.2017.09.013.

151. Saito, H., Tanaka, I., Akahira, J.-i., Endo, M., Igarashi, K., Okuzono, T., et al. (2023). Muscular metastasis of superficial esophageal squamous cell carcinoma: a rare case of recurrence after endoscopic submucosal dissection with additional chemoradiotherapy. *Clinical Journal of Gastroenterology, 16*(2), 130-135, doi:10.1007/s12328-022-01735-0.

152. Sakamoto, Y., Yokouchi, M., Nagano, S., Shimada, H., Nakamura, S., Setoguchi, T., et al. (2014). Metastasis of osteosarcoma to the trapezius muscle: a case report. *World Journal of Surgical Oncology, 12*(1), 1-6, doi:10.1186/1477-7819-12-176.

153. Salar, O., Flockton, H., Singh, R., & Reynolds, J. (2012). Piriformis muscle metastasis from a rectal polyp. *Case Reports, 2012*, bcr2012007208, doi:10.1136/bcr-2012-007208.

154. Salemis, N. S. (2015). Skeletal muscle metastasis from breast cancer: management and literature review. *Breast Disease, 35*(1), 37-40, doi:10.3233/BD-140384.

155. Salman, R., Sebaaly, M. G., Asmar, K., Nasserdine, M., Bannoura, S., & Khoury, N. J. (2018). Rare skeletal muscle metastasis from renal cell carcinoma: case report and review of the literature. *CEN case reports, 7*(2), 316-319, doi:10.1007/s13730-018-0350-1.

156. Sanguinetti, A., Bistoni, G., Calzolari, F., Lucchini, R., Monacelli, M., Triola, R., et al. (2012). Cystosarcoma phyllodes with muscular and lymph node metastasis. *Ann. Ital. Chir, 1*(4), 331-336.

157. Sariaydin, M., Günay, E., Ulasli, S. S., Günay, S., Yavaş, B. D., Tokyol, Ç., et al. (2016). An unusual metastasis of lung adenocarcinoma: Biceps brachii muscle. *Lung India: Official Organ of Indian Chest Society, 33*(6), 669, doi:10.4103/0970-2113.192857.

158. Sarma, M., Sonik, B., Subramanyam, P., & Sundaram, P. S. (2015). Isolated skeletal muscle metastatic deposit in a patient with micropapillary carcinoma thyroid identified by 18F FDG PET CT. *Journal of the Egyptian National Cancer Institute, 27*(1), 47-50, doi:10.1016/j.jnci.2015.01.002.

159. Savvidou, O. D., Chloros, G. D., Agrogiannis, G. D., Korkolopoulou, P., Panagopoulos, G. N., & Papagelopoulos, P. J. (2016). Skeletal muscle metastasis of a GIST: a case report and review of the literature. *Case reports in surgery, 2016*, 7867545, doi:10.1155/2016/7867545.

160. Sellami, M., Ayadi, S., Abbes, A., Mnejja, M., Hammami, B., Boudaouara, T., et al. (2022). Diplopia Secondary to Gastric Adenocarcinoma Metastasis to the Superior Oblique Muscle. *Ear, Nose Throat Journal*, 01455613221145277, doi:10.1177/01455613221145277.

161. Sharma, V., Bhardwaj, R., Chaudhary, K., Pandey, M., & Sharma, S. (2015). A case of metastatic infiltrating ductal breast carcinoma with initial metastases to the optic nerve and subsequent extra ocular muscle involvement. *Indian journal of cancer, 52*(3), 275-276, doi:10.4103/0019-509X.176757.

162. Shaukat, U., Rehman, U., Younis, M. N., & Shahid, A. (2021). Malignant Phyllodes Breast Tumor: A Rare Case of Metastasis in Skeletal Muscle Detected on 18-Fluorodeoxyglucose Positron Emission Tomography. *Cureus, 13*(5), e15274, doi:10.7759/cureus.15274.

163. Shetty, N. S., Calhoun, A., Sunjaya, D., Greer, A., & Willingham, F. F. (2021). Retroperitoneal fibrosis with skeletal muscle invasion as an early manifestation of metastatic gastric cancer. *ACG case reports journal, 8*(4), doi:10.14309/crj.0000000000000553.

164. Shi, L. N., Qiu, Z. L., Wu, C. G., & Luo, Q. Y. (2015). A rare case of calf muscle metastasis from a non-functional pancreatic neuroendocrine carcinoma. *Iranian Journal of Radiology, 12*(2), e11637, doi:10.5812/iranjradiol.11637.

165. Shimizu, K., Hashimoto, D., Umezaki, N., Nakagawa, S., Yamamura, K., Chikamoto, A., et al. (2018). Thoracic wall muscle metastasis from pancreatic cancer. *Surgical Case Reports, 4*(1), 12, doi:10.1186/s40792-017-0393-0.

166. Shupp, B., Liaquat, H., Prenatt, Z., Stoll, L., & Matin, A. (2023). A Rare Case of Abdominal Wall Skeletal Muscle Metastasis From Adenocarcinoma of the Pancreatic Head. *Cureus, 15*(7), e41470, doi:10.7759/cureus.41470.

167. Sim, W. Y., Park, N. H., & Kwon, T. J. (2021). Unusual sonographic appearance of breast cancer metastasis to the pectoralis muscle. *Journal of Clinical Ultrasound, 49*(8), 881-884, doi:10.1002/jcu.23042.

168. Skenderi, F., Chikha, A., Ibisevic, N., Tatarevic-Suko, A., Kantardzic, N., & Vranic, S. J. I. J. o. G. P. (2017). Skeletal muscle metastases from squamous cell carcinoma of the cervix: report of two cases with literature review. *Int J Gynecol Pathol, 36*(1), 95-100, doi:10.1097/PGP.0000000000000298.

169. Spetsieris, N., Bobba, G., Bhandari, P., Patel, S., Tharayil, Z., & Gupta, R. (2022). Pancreatic Cancer with a Skeletal Muscle Metastasis-A Case Presentation and Literature Review. *Journal of Community Hospital Internal Medicine Perspectives, 12*(5), 60, doi:10.55729/2000-9666.1092.

170. Spinelli, M., & Gillibrand, R. (2018). Metastasis to gluteal muscle from high grade transitional cell carcinoma of bladder. Report of a case and review of literature. *Pathologica, 110*(1), 78-81.

171. Stergioula, A., Pantelis, E., Kormas, T., & Agrogiannis, G. (2023). Case report: skeletal muscle metastasis from follicular thyroid carcinoma presenting as synovial sarcoma. *Frontiers in Oncology, 13*, 994729, doi:10.3389/fonc.2023.994729.

172. Strauss, J. B., Shah, A. P., Chen, S. S., Gielda, B. T., & Kim, A. W. (2012). Psoas muscle metastases in non-small cell lung cancer. *Journal of thoracic disease, 4*(1), 83, doi:10.3978/j.issn.2072-1439.2011.04.05.

173. Suhr, A., Choi, J. J., & Levine, T. C. (2022). Cutaneous Melanoma with Metastasis to the Lateral Rectus Muscle. *Ophthalmology, 129*(9), 1021, doi:10.1016/j.ophtha.2022.03.002.

174. Suzuki, K., Yasuda, T., Nagao, K., Hori, T., Watanabe, K., Kanamori, M., et al. (2014). Metastasis of gastrointestinal stromal tumor to skeletal muscle: a case report. *Journal of Medical Case Reports, 8*(1), 1-5, doi:10.1186/1752-1947-8-256.

175. Takahashi, K., Putchakayala, K. G., Safwan, M., & Kim, D. Y. (2017). Extrahepatic metastasis of hepatocellular carcinoma to the paravertebral muscle: A case report. *World journal of hepatology, 9*(22), 973, doi:10.4254/wjh.v9.i22.973.

176. Taniguchi, K., Susa, M., Ogata, S., Ozeki, Y., & Chiba, K. (2017). Thymoma Metastasis to the Semimembranosus Muscle. *Case reports in oncology, 10*(1), 21-26, doi:10.1159/000455190.

177. Tatsuta, K., Harada, T., & Nishiwaki, Y. (2022). Cervical Skeletal Muscle Metastasis of Colorectal Cancer. *Internal Medicine, 61*(2), 263-264, doi:10.2169/internalmedicine.7817-21.

178. Tessi, R. T. Y., Adeyemi, B. A., El Aoufir, O., Jerguigue, H., Latib, R., & Omor, Y. (2021). Muscle involvement by widely metastatic squamous cell carcinoma of the lung. *Radiology Case Reports, 16*(7), 1836-1839, doi:10.1016/j.radcr.2021.04.037.

179. Tezcan, Y., & Koc, M. (2014). Muscle metastasis from non-small cell lung cancer: two cases and literature review. *Acta Clinica Belgica, 69*(4), 302-304, doi:10.1179/2295333714Y.0000000035.

180. Thumallapally, N., El-Bitar, S., Mohammad, F., & Atallah, J. P. (2018). Extraocular muscle metastasis from esophageal carcinoma: an atypical and rare presentation. *Journal of gastrointestinal cancer, 49*(2), 211-213, doi:10.1007/s12029-016-9887-4.

181. Togral, G., Arıkan, M., & Gungor, S. (2014). Rare skeletal muscle metastasis after radical nephrectomy for renal cell carcinoma: evaluation of two cases. *Journal of Surgical Case Reports, 2014*(10), rju101, doi:10.1093/jscr/rju101.

182. Tunio, M. A., AlAsiri, M., Riaz, K., AlShakwer, W., & AlArifi, M. (2013). Skeletal muscle metastasis as an initial presentation of follicular thyroid carcinoma: a case report and a review of the literature. *Case Reports in Endocrinology, 2013*, doi:10.1155/2013/192573.

183. Tuysuz, G., Özdemir, N., Emir, H., Durak, H., Dervisoglu , S., Adaletli, I., et al. (2017). A Translocation Renal Cell Carcinoma with Skeletal Muscle Metastasis in a Child. *Turkish Journal of Pathology, 33*(3), 248-250, doi:10.5146/tjpath.2014.01259.

184. Urgun, D. A., Bista, B., & Krishnam, M. (2020). Unusual presentation of testicular rhabdomyosarcoma metastases to the R. ventricle papillary muscle and tricuspid valve. *Radiology Case Reports, 15*(9), 1562-1565, doi:10.1016/j.radcr.2020.05.075.

185. Uygur, S., Aral, M., Kaya, B., Ozmen, S., Ozgun, G., & Latifoglu, O. (2011). Giant temporalis muscle metastasis of esophageal carcinoma. *Journal of Craniofacial Surgery, 22*(2), 736-737, doi:10.1097/SCS.0b013e318208bae9.

186. Valkenborgh, C., Médart, L., & Collignon, L. (2019). Muscle metastasis from undifferentiated (anaplastic) thyroid carcinoma. *Journal of the Belgian Society of Radiology, 103*(1), 27, doi:10.5334/jbsr.1604.

187. Varadarajan, I., Basu, A., Besmer, S., Poli, J., Richard, S., & Styler, M. (2017). Solitary skeletal muscle metastasis as first site of recurrence of cervical cancer: a case report. *Case reports in oncology, 10*(2), 694-698, doi:10.1159/000478976.

188. Vijay, K., Ashish, C., Kabilan, C., Heng, Y. W., & Peh, W. C. (2017). Unusual presentation of lung cancer as skeletal muscle and subcutaneous metastases. *Lung India: Official Organ of Indian Chest Society, 34*(5), 485, doi:10.4103/0970-2113.213820.

189. Vishnoi, M. G., Jain, A., John, A. R., & Paliwal, D. (2016). A rare case of extensive skeletal muscle metastases in adenocarcinoma cervix identified by 18F-fluorodeoxyglucose positron emission tomography/computed tomography scan. *Indian Journal of Nuclear Medicine: IJNM: The Official Journal of the Society of Nuclear Medicine, India, 31*(3), 215, doi:10.4103/0972-3919.183609.

190. Vu, L., & Haygood, T. M. (2014). Primary sacrococcygeal chordoma with unusual skeletal muscle metastasis. *Radiology Case Reports, 9*(4), 921, doi:10.2484/rcr.v9i4.921.

191. Vural, A., Avcı, D., Çağlı, S., Yüce, İ., & Arlı, T. (2017). Gluteus medius muscle metastasis of squamous cell carcinoma of larynx: a rare case. *Brazilian journal of otorhinolaryngology, 86 Suppl 1*(Suppl 1), 23-25, doi:10.1016/j.bjorl.2017.04.002.

192. Warren, C. C., Liao, J. C., & Griepentrog, G. J. (2016). Bilateral extraocular muscle metastases from a gastrointestinal carcinoid tumor. *JAMA ophthalmology, 134*(1), e153651-e153651, doi:10.1001/jamaophthalmol.2015.3651.

193. Weeraddana, P., Walgamage, T., Elkabbani, R., Dmitriev, M., Crespo-Quezada, J., Dandwani, M., et al. (2023). An Unusual Case of Lung Adenocarcinoma Metastasis to the Tricep Muscle: Four Years Disease-Free After Surgical Resection and Radiotherapy. *Cureus, 15*(4), e38347, doi:10.7759/cureus.38347.

194. Xue, Y.-L., Song, H.-J., Qiu, Z.-L., & Luo, Q.-Y. (2014). Large thigh and buttock muscle metastases as the initial manifestation of follicular thyroid cancer. *Clinical nuclear medicine, 39*(4), 363-364, doi:10.1097/RLU.0b013e3182a75589.

195. Yang, J., Li, L.-F., Zhang, X.-M., Xu, Q., Zhang, J., Weng, W.-W., et al. (2015). Unusual synchronous skeletal muscle and lung metastasis in papillary thyroid cancer: A case report and review of the literature. *Oncology letters, 9*(2), 727-730, doi:10.3892/ol.2014.2742.

196. Yilmaz, M., Elboga, U., Celen, Z., Isik, F., & Tutar, E. (2011). Multiple muscle metastases from lung cancer detected by FDG PET/CT. *Clinical nuclear medicine, 36*(3), 245-247, doi:10.1097/RLU.0b013e318208f75d.

197. Yurkiewicz, I. R., Ganjoo, K. N., & Iagaru, A. (2018). Anaplastic Thyroid Cancer With Extensive Skeletal Muscle Metastases on 18F-FDG PET/CT. *Clinical nuclear medicine, 43*(4), e113-e114, doi:10.1097/RLU.0000000000001968.

198. Zhang, Z., Guo, S., Zuo, C., & Cheng, C. (2023). A Case of Pancreatic Cancer With Rare Cutaneous and Muscle Metastases on 18F-FDG PET/CT Imaging. *Clinical nuclear medicine, 48*(8), 692-693, doi:10.1097/Rlu.0000000000004699.

199. Zhao, L.-x., Li, L., Li, F.-l., & Zhao, Z. (2010). Rectus abdominis muscle metastasis from papillary thyroid cancer identified by I-131 SPECT/CT. *Clinical nuclear medicine, 35*(5), 360-361, doi:10.1097/RLU.0b013e3181d6265b.

200. Zhong, W., You, C., Chen, H., & Huang, S. (2012). Primary presacral carcinoid tumor with gluteal muscle metastasis. *Neurology India, 60*(5), 544, doi:10.4103/0028-3886.103219.

201. Zhu, G., Sun, W., Liu, Y., Wang, H., & Ye, S. (2021). Skeletal muscle metastasis from a gastrointestinal stromal tumor: A case report. *Medicine, 100*(34), e27011, doi:10.1097/MD.0000000000027011.
